# Morphological and Molecular Characterization of *Trichoderma* Isolates from Vegetable Crop Rhizospheres in Nepal

**DOI:** 10.12688/f1000research.153701.3

**Published:** 2025-03-05

**Authors:** Puja Jaiswal, Ram B. Khadka, Aashaq Hussain Bhat, Suraj Baidya, Arvind Kumar Keshari

**Affiliations:** 1 Central Department of Zoology, Tribhuvan University, Kirtipur, Kathmandu, Bagmati, 0097, Nepal; 2 National Plant Pathology Research Centre, Nepal Agricultural Research Council, Lalitpur, Bagmati, 5459, Nepal; 3 Department of Bioscience, Chandigarh University, Sahibzada Ajit Singh Nagar, Punjab, India; 4 Department of Zoology, Patan Multiple Campus, Tribhuvan University, Lalitpur, Bagmati, 0097, Nepal

**Keywords:** Biocontrol agent; phylogenetic analysis; Rhizospheric region; Species diversity; Nepal

## Abstract

**Background:**

*Trichoderma* spp. hold significant potential as biocontrol agents in agriculture due to their antagonistic properties against plant pathogens. The study aimed to characterize and identify *Trichoderma* isolates from rhizospheric regions of vegetable crops.

**Methods:**

In this study, *Trichoderma* isolates were collected from rhizospheric soil samples of vegetable crops from different ecological zones and were selected for comprehensive morphological and molecular characterization. The isolates were visually assessed for colony color, growth pattern, aerial mycelium presence, phialide and conidial morphology, and chlamydospore presence. Molecular analysis was employed based on ITS and tef-1α sequences. Diversity indices were also computed for different ecological zones.

**Results:**

The morphological characteristics and phylogenetic trees for both regions provided a clear species resolution, with four main clades: *Harzianum, Viride, Brevicompactum* and *Longibrachiatum* with 12 species *T. harzinaum*, *T. afroharzianum, T. lentiforme, T. inhamatum, T. camerunense, T. azevedoi*, *T. atroviride, T. asperellum, T. asperelloides, T. koningii, T. longibrachiatum* and *T. brevicompactum* and nine species as a new country record. Diversity indices indicated that high mountain regions displayed the highest species diversity and evenness (H = 1.724 [0.28], J = 0.84, D = 0.28), followed by hilly regions (H = 1.563 [0.28], J = 0.72, D = 0.28). Plains, on the other hand, exhibited lower species diversity (H = 1.515, J = 0.66, D = 0.33). The calculated species abundance values showed that plains (E = 2.11), mid-hills (E = 1.95), and high mountains (E = 1.99) each had their unique diversity profiles. Notably, *T. afroharzianum* and *T. asperellum* were predominant.

**Conclusions:**

Overall, the study unveiled a rich diversity of *Trichoderma* species in different agricultural zones of Nepal. These findings shed light on the ecological distribution and diversity of *Trichoderma* spp., which could have significant implications for sustainable agriculture and biological control strategies.

## Introduction

The genus *Trichoderma* comprises a highly diverse group of soil dwelling fungi, demonstrating remarkable versatility. These fungi are known for their rapid growth and adaptability, colonizing various habitats from forest soils to agricultural fields. Within this genus, a wide array of strains exhibits significant potential for various applications, including biological control as biopesticides, biofertilizers, soil enhancers and promoters of crop growth. ^1^^,^^2^ Furthermore, *Trichoderma* isolates have the capacity to stimulate plant defense mechanisms, a phenomenon extensively documented in studies conducted by various researchers. ^3^^–^^6^

The *Trichoderma* genus was originally proposed by Persoon in Germany in 1794, more than two centuries ago, and their potential as bioagents was first documented by Weindling in 1932. ^7^ To date, over 250 *Trichoderma* species have been identified in soil environments worldwide. ^8^ *Trichoderma* species can be readily isolated from soil using various conventional methods, thanks to their adaptability to diverse conditions, ranging from deserts and wetlands to the Himalayan regions. However, the most effective method for isolating *Trichoderma* spp. is from rhizospheric soil samples. ^9^ A comprehensive understanding of *Trichoderma* biodiversity is crucial for harnessing the full potential of this genus in various biological applications. Previous surveys that investigated *Trichoderma* diversity in specific geographical regions have primarily focused on isolates obtained from soil samples. These surveys have been conducted in various locations, including Russia and the Siberian Himalayas, ^10^^,^^11^ Cerrado, ^12^ China, ^13^^,^^14^ South-East Asia, ^15^ India, ^16^ Egypt, ^17^ Iran, ^18^ Philippines, ^19^ Europe, ^20^ Canary Islands, ^21^ Sardinia, ^22^ South America, ^23^ and Turkey. ^24^ The potential of *Trichoderma* species as biological control agents (BCAs) has been under investigation for over 70 years, and more recently, these species have become commercially available. ^25^^,^^26^ Certain *Trichoderma* species have been successfully utilized as BCAs, showcasing their antagonistic mechanisms to mitigate damage caused by soil-borne pathogens, both in greenhouse and open field conditions. ^27^^–^^31^ *Trichoderma* deploys diverse mechanisms, including antibiosis, competition for nutrients and space, mycoparasitism, production of antimicrobial compounds, induction of plant defense responses and enzymatic hydrolysis, to manifest its biocontrol activity. ^32^ These capabilities not only help in managing diseases but also promote plant growth and resilience.

Nepal’s agriculture is dominated by smallholder farmers with limited access to modern agricultural inputs such as chemical pesticides and fertilizers. The country’s diverse agro-ecological zones present both opportunities and challenges for crop production. *Trichoderma*, with its versatile role in crop production, offers eco-friendly and cost-effective solutions to challenges like biotic stresses (diseases and pests) and abiotic stresses (drought and cold). Additionally, it addresses issues of low soil fertility, particularly in hilly regions, by strengthening plant root systems to improve nutrient and soil absorption. Therefore, identifying native *Trichoderma* isolates that are better adapted to the local environment, thrive better in local soil conditions, and exhibit versatile capabilities in suppressing multiple stresses in crops is critically important for the successful commercial application of *Trichoderma* in Nepal. The native isolates of *Trichoderma* can effectively suppress soil-borne pathogens and improve plant health. Despite the recognized benefits, the commercialization and widespread adoption of *Trichoderma*-based products in Nepal remain limited. While specific data on the quantity of commercial *Trichoderma* products and their usage in Nepal are scarce, efforts are underway to explore and promote their application. For instance, initiatives like the “Initial Scoping for Commercial Marketing of *Trichoderma* and other Biological Solutions in Nepal” aim to assess and enhance the market potential of these biocontrol agents. However, it is important to note that not all *Trichoderma* species yield the same effects on crops or pathogens. Given the variability in efficacy among different *Trichoderma* species and strains, it is crucial to identify and characterize native isolates that are well-adapted to local conditions for their effective application as BCAs and the preservation of isolates as viable commercial products. ^26^ Such tailored approaches can maximize the benefits of *Trichoderma* applications, offering eco-friendly and cost-effective solutions to the challenges faced by Nepalese farmers. Traditional methods for identifying *Trichoderma* species have conventionally relied on morphological characteristics and culture-based approaches. However, owing to the limited and often indistinguishable morphological features shared among *Trichoderma* species, alongside the increasing recognition of morphologically cryptic species, accurate identification through these methods becomes challenging and can potentially lead to misidentification. ^10^^,^^33^ As a result, molecular techniques such as amplification of important taxonomic markers, and sequencing have emerged as indispensable tools for achieving precise species identification. ^33^^–^^36^ In the present study, the identification of *Trichoderma* species at the species level was accomplished by focusing on two key genetic markers: the Internal Transcribed Spacer (ITS) region of rDNA and a partial sequence of the translation elongation factor 1-alpha gene (tef-1α) in addition to classical taxonomy. ^37^ Recent researches have evaluated the ecological specialization of diversity. Numerous studies have documented the discovery of new isolates and phylogenetic species across various natural ecosystems. ^21^^,^^38^ While some studies have been conducted in Nepal, they all have focused on different ecological zones. ^39^ In contrast, limited attention has been given to studying *Trichoderma* in agricultural environments. Importantly, such investigations have practical implications, as they highlight the potential of agricultural soil rhizosphere as a rich source of beneficial strains with biocontrol capabilities. Building upon these findings, we propose that the composition, distribution, and genetic structure of *Trichoderma* spp. in the rhizosphere of vegetable crops along with ecological domains and altitudinal gradients may exhibit significant differences. Confirming these differences will enhance our understanding of *Trichoderma*’s biodiversity across distinct biological niches and various altitudinal gradients across the country.

## Methods

### Isolation and culture of *Trichoderma* isolates

**Figure 1.**
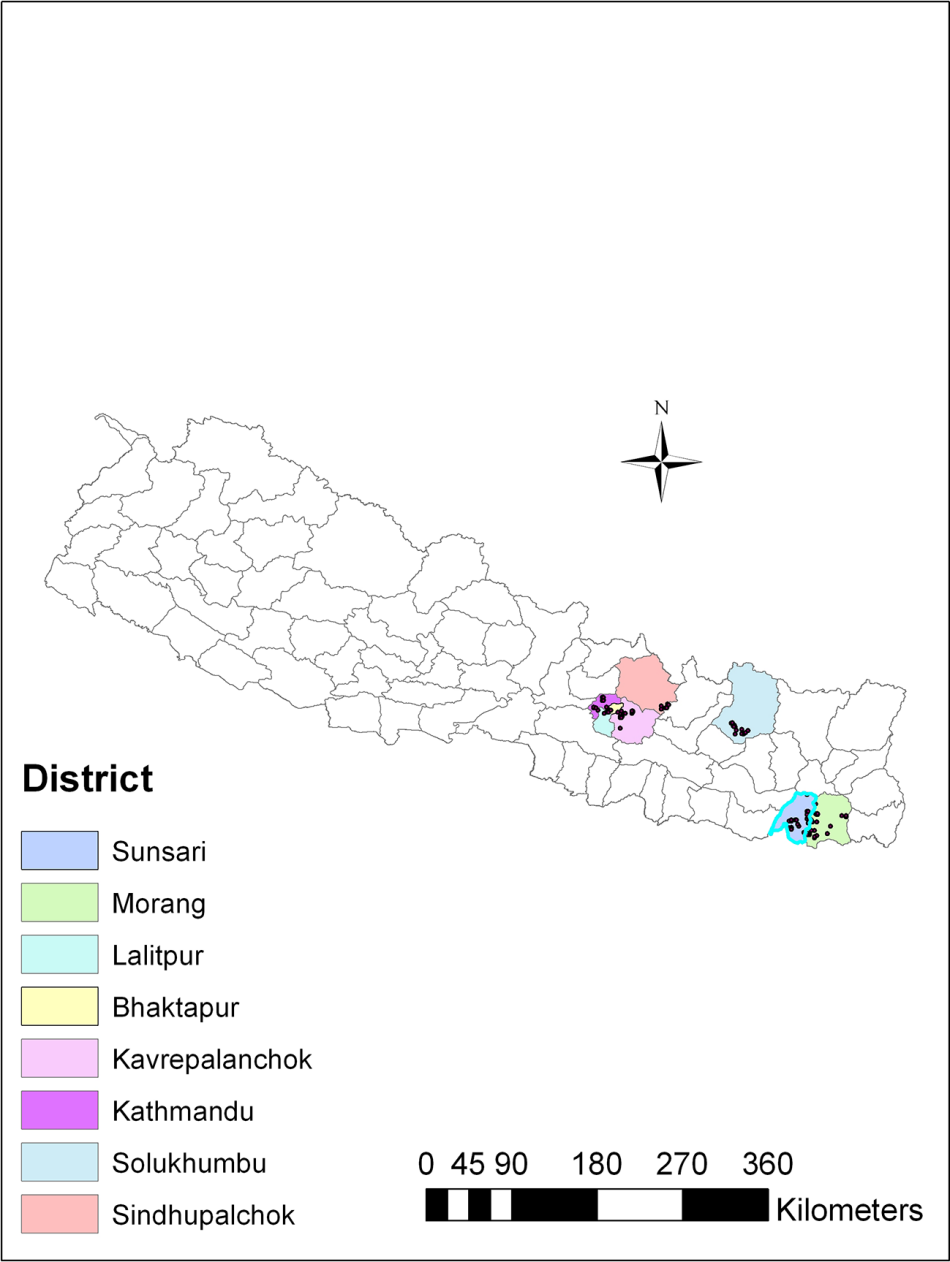
Map showing soil sampling sites for isolation of *Trichoderma* isolates.

To isolate *Trichoderma*, soil samples were collected from different agricultural regions spanning three ecological zones in Nepal: the plain region (80-141 m above sea level), the mid-hill region (1284-1650 m above sea level), and the high mountain region (1800-2500 m above sea level). These regions exhibited variations in altitude and ecological traits, and were recognized for cultivating a range of agricultural crops. The soil samples were collected from vegetable-growing areas within each region, covering eight districts in Nepal, including Sindhupalchok and Solukhumbu districts in the high mountains; Kathmandu, Lalitpur, Bhaktapur and Kavrepalanchok districts in the mid-hills, and Sunsari and Morang in the plains ( Figure 1).

Using a sterile spatula, approximately 200 grams of soil were collected from the rhizospheric zone of crops such as cauliflower, leaf mustard, cabbage, asparagus bean, tomato, cucumber, brinjal, chilly, pumpkin, and okra. These soil samples were carefully placed in clean polythene bags and appropriately labelled. Subsequently, the soil samples were transported to the National Plant Pathology Research Center, Khumaltar, Nepal and stored at 4°C until isolation.

For isolation, 10 grams soil samples were suspended in 90 ml of sterile distilled water and mixed using a rotary shaking machine set at 260 × g for 30 minutes to ensure thorough homogenization. ^40^ To facilitate the isolation, 1 mL aliquots of the soil suspensions were diluted from 10 ^−1^ to 10 ^−5^ onto *Trichoderma* Selective Media (TSM) using the spread plate technique as described by Askew and Laing. ^41^ After inoculation, *Trichoderma* colonies were sub-cultured onto potato dextrose agar (PDA) plates and incubated at 28±2°C for seven days to allow for growth. Colonies have key characteristics that can be used to identify them as *Trichoderma* including growth pattern, growth speed, and colour. Depending on the diversity of colony characteristics, one to two colonies were selected from each sample for pure culture preparation. Each individual isolate was assigned a unique code and stored in a PDA slant for further study. Isolates were then grown on PDA plates for 3-5 days for sporulation. Different dilutions (10 ^−1^ to 10 ^−3^) of spore suspension for each isolate were spread on 10% water agar plates and incubated at 28±2°C for 24 hours in the dark to prepare monoconidial cultures. A germinating, isolated single spore was picked up using a sterile needle from the water agar plates and placed on PDA plates to prepare monoconidial culture for each isolate. Spore suspensions were prepared as mentioned earlier and preserved in silica gel for long term storage at -80°C in a deep freezer. ^42^

### Morphological and morphometrical characterization

A total of 167 *Trichoderma* isolates were isolated from various soil samples and preserved using silica gel and were subsequently cultured on PDA plates at 28±2°C for three days. Following the incubation period, cultures with similar morphological characteristics, including growth pattern, diffusible pigments and odor, were observed and sorted into 25 different groups (T1-T25). While most groups consisted of 4 to 5 isolates, T11 and T22 (two larger groups) comprised 20 to 25 isolates each. Within each group, one representative isolate was selected for further study, except for the larger groups where 3 to 5 isolates were chosen for the study. Morphological characteristics such as the aspects of phialides, conidia and chlamydospores were scrutinized under a light microscope (Optika Microscope Italy, B-383PLi) equipped with a camera. To achieve this, *Trichoderma* isolates were grown on corn meal agar plate for 2-3 days, after which mycelial tips from the colony margin were transferred onto microscope slide and mounted with 3% KOH using sterile glass needles. Subsequently, these specimens were subjected to observation following the flattening and stretching of hyphae with a pair of glass needles. Once suitably prepared, a coverslip was placed over the samples, and any excess liquid was removed using tissue paper. Microscopical measurements and analyses were conducted using the ImageJ software ( https://imagej.en.download.it, LOCI, University of Wisconsin). A total of 30 individual phialides and conidia from each specimen were measured and analyzed.

The colony characteristics of *Trichoderma* isolates were investigated on PDA plates that were incubated at 28°C under dark conditions. Notably, all *Trichoderma* isolates developed conidia within 4-5 days. Various diffusible pigments were observed, manifesting as distinct colors, which were then compared with the Audrey & Bear Color Chart ( https://audreyandbear.com/products/audrey-bear-color-chart). Additionally, the presence or absence of a coconut odor was noted following 48 hours of culturing on PDA plates. ^43^

### Molecular characterization of *Trichoderma* isolates

**Figure 2.**
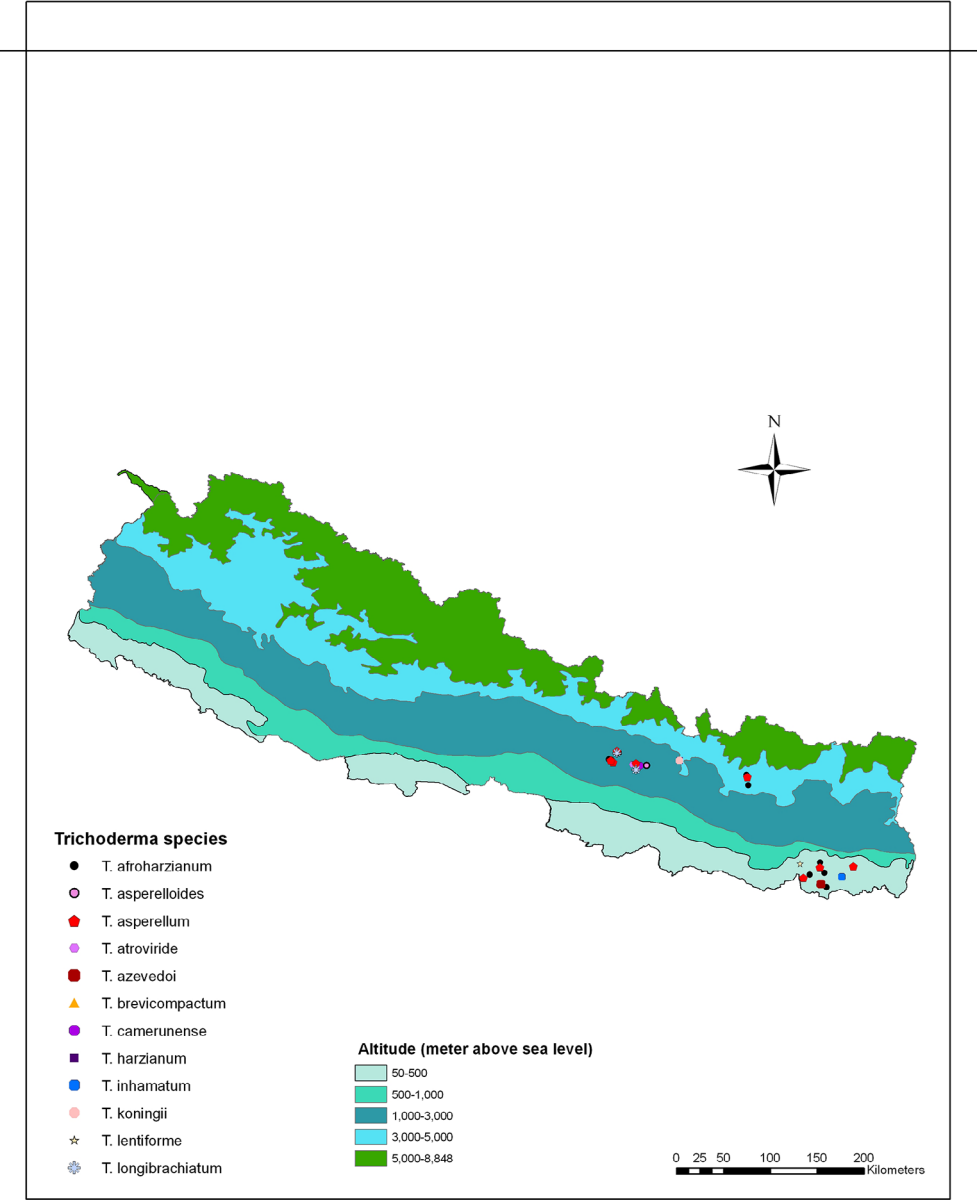
Distribution of *Trichoderma* species/isolates collected across various ecological regions of Nepal.

Thirty-three *Trichoderma* spp. isolates, representing diverse groups based on similarities in morphological traits such as growth patterns and diffusible pigments, were selected from various ecological regions for DNA extraction and molecular characterization ( Figure 2).

Approximately, 30-40 mg of mycelium was scraped off from 3–5-day-old colonies cultured on potato dextrose agar (PDA) using sterilized glass slides prior to sporulation for genomic DNA extraction. The obtained mycelia were then ground in liquid nitrogen and DNA extraction was carried out using the Promega Wizard genomic DNA extraction kit (Catalogue No. A1120) following the manufacturer’s instructions (Promega Corporation, Madison, WI).

A fragment of rRNA containing the internal transcribed spacer regions was amplified using the primers ITS4 (TCCTCCGCTTATTGATATGC) and ITS5 (GGAAGTAAAAGTCGTAACAAGG). ^44^ The translation elongation factor 1-alpha gene (tef-1α) was amplified using the primers EF1-728F (CATCGAGAAGTTCGAGAAGG) ^45^ and TEF1R (GCCATCCTTGGGAGATACCAGC). ^10^^,^^46^^,^^47^ Each PCR reaction (25 μL) comprised 12.5 μL of GoTaq ^®^ Green Master Mix (Promega), 9.5 μL of nuclease-free water, 1 μL of each forward and reverse primer (10 mM), and 1 μL of gDNA. The PCR reactions were performed using a thermocycler (LifeEco Thermal Cycler (BIOER)) with the following settings: For ITS: 1 cycle of 2 minutes at 95°C followed by 34 cycles of 30 seconds at 95°C, 30 seconds at 57°C, 1 minute at 72°C, followed by a final elongation step at 72°C for 5 minutes. For tef-1α: 1 cycle of 5 minutes at 95°C followed by 35 cycles of 45 seconds at 95°C, 45 seconds at 63°C, 1 minute at 72°C, followed by a final elongation step at 72°C for 10 minutes. Amplified PCR products (3 μL) were subjected to electrophoresis (45 minutes, 100 volts) in a 1.0% agarose gel stained with GelGreen ^®^ Nucleic Acid Gel Stain (Biotium, Fremont, CA, USA) alongside a 1 kb DNA ladder (Promega) for size estimation of the amplified bands. The PCR products were purified using the Wizard SV gel and PCR cleanup system (Promega) following the manufacturer’s instructions and were subsequently sequenced using both reverse and forward primers by Sanger sequencing (BGI Solutions Co., Ltd. Tai Po, Hong Kong, China).

### Phylogenetic relationships

Sequences were manually edited using Finch TV 1.4.0 for Windows (Geospiza, https://digitalworldbiology.com/FinchTV). Consensus sequences of forward and reverse amplicons were created using BioEdit software ^48^ and deposited in the National Center for Biotechnology Information (NCBI) NCBI under accession numbers (OR140790- OR140818) for ITS rRNA and (OR567133-OR567158) for tef-1α. The BLASTn similarity search program was employed to identify homologous sequences in the NCBI nucleotide database, confirming species-level similarity with the sequence of the isolates. The ITS and tef-1α gene sequences were concatenated using Mesquite software version 2.75. ^49^ Final alignments were done using Clustal W 2.0 in MEGA X version 10.1 and were used to construct phylogenetic trees using the Maximum Likelihood method with the Kimura 2-parameter (G+I for ITS) and Hasegawa-Kishino-Yano (G+I for tef-1α) models. ^50^ The choice of the evolution model was made after evaluating individual alignments through a model test (MEGAX software). Bootstrap values were computed with 1,000 replications to assess the statistical support for each branch. Graphical representation and editing of the phylogenetic trees were carried out using the Interactive Tree of Life (v3.5.1). ^51^^,^^52^

**Analysis of the diversity among** ***Trichoderma*** **species**

The degree of dominance index, also known as the McNaughton dominance index (Y), was used to quantitatively assess the habitat preference of *Trichoderma* isolates in different agricultural fields. The dominance values were calculated using the equation:

$$Y=\frac{\mathrm{ni}\;}{\mathrm{fiN}\;}$$

where ‘N’ is the total number of *Trichoderma* isolates, ‘ni’ is the number of the genus (species) i, and ‘fi’ is the frequency with which genus (species) i appears in the samples. A species i is considered dominant when Y > 0.02. ^53^

Species richness (the total number of species), abundance (the total number of isolates of each species), and diversity were calculated using several ecological indices: the Simpson biodiversity index (D), ^54^ Shannon’s biodiversity index (H), ^55^ Pielou species evenness index (J), ^56^ and Margalef’s abundance index (E). ^57^ These indices were applied to quantitatively describe the diversity and habitat preferences of *Trichoderma* species in various agricultural fields across different ecological zones of Nepal.

The biological diversity indices were computed numerically using the following equations in Microsoft Excel (Excel 2010 (v14.0))

$$D=1-\frac{\Sigma \mathrm{ni}\left(\mathrm{ni}-1\right)\;}{N\left(N-1\right)\;}$$

H=ΣPilnPi

where Ni = 1, and Pi *=* $\frac{\mathrm{ni}}{N\;}$

$$J=\frac{H}{\text{Hmax}\;}$$

Where Hmax = InS

$$E=\frac{S-1\;}{\ln\;N\;}$$

In these equations, ‘S’ is the total number of *Trichoderma* species, ‘N’ is the total number of *Trichoderma* species isolates, ‘Pi’ is the relative quantity of *Trichoderma* species ‘i’, and ‘ni’ is the number of isolates of *Trichoderma* species ‘i’.

## Results

### Morphological characterization of single spores of *Trichoderma* isolates

**Plate 1.**
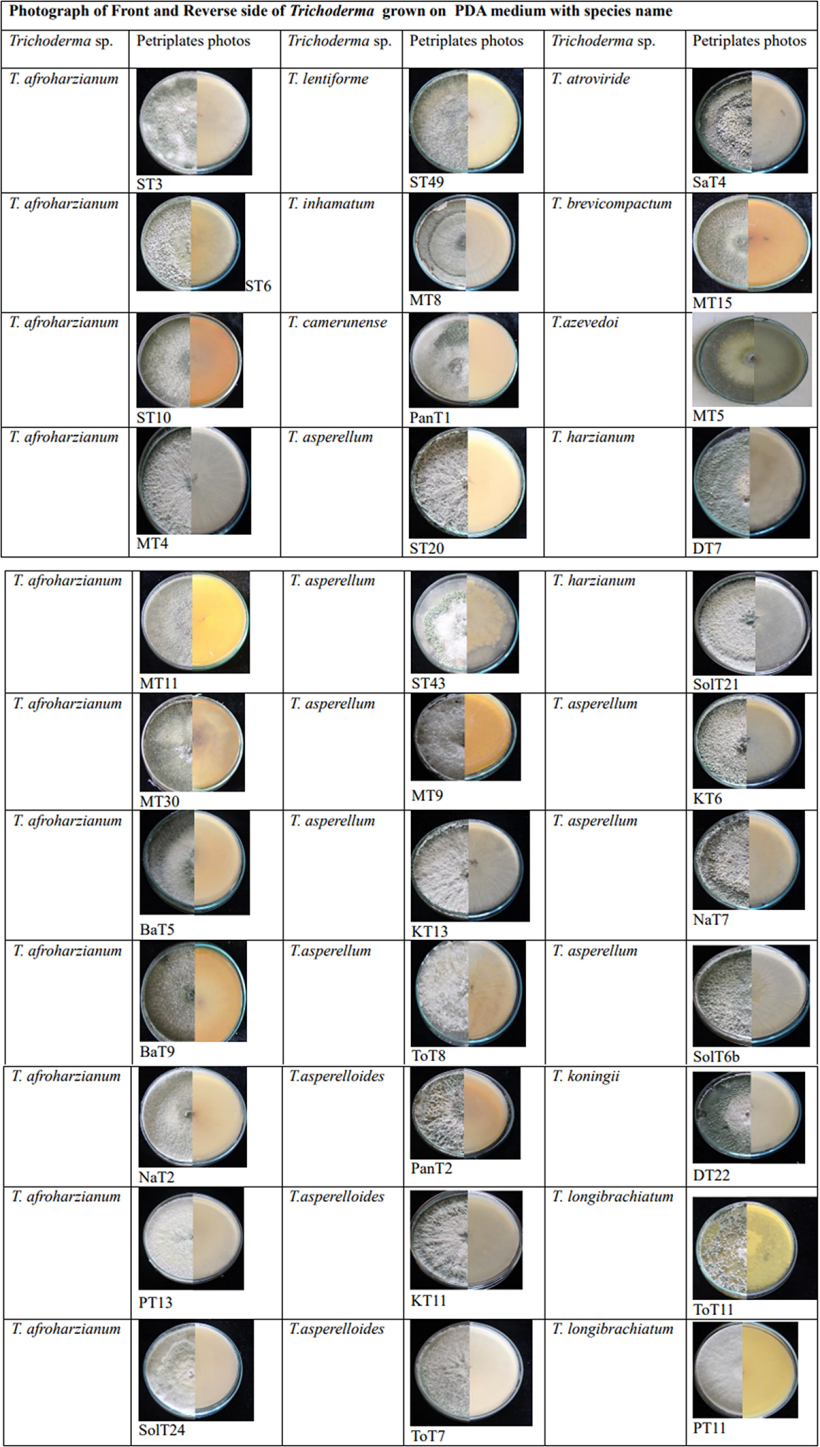
Culture of *Trichoderma* isolates grown in 28±2°c in potato dextrose agar medium.

**Figure 3.**
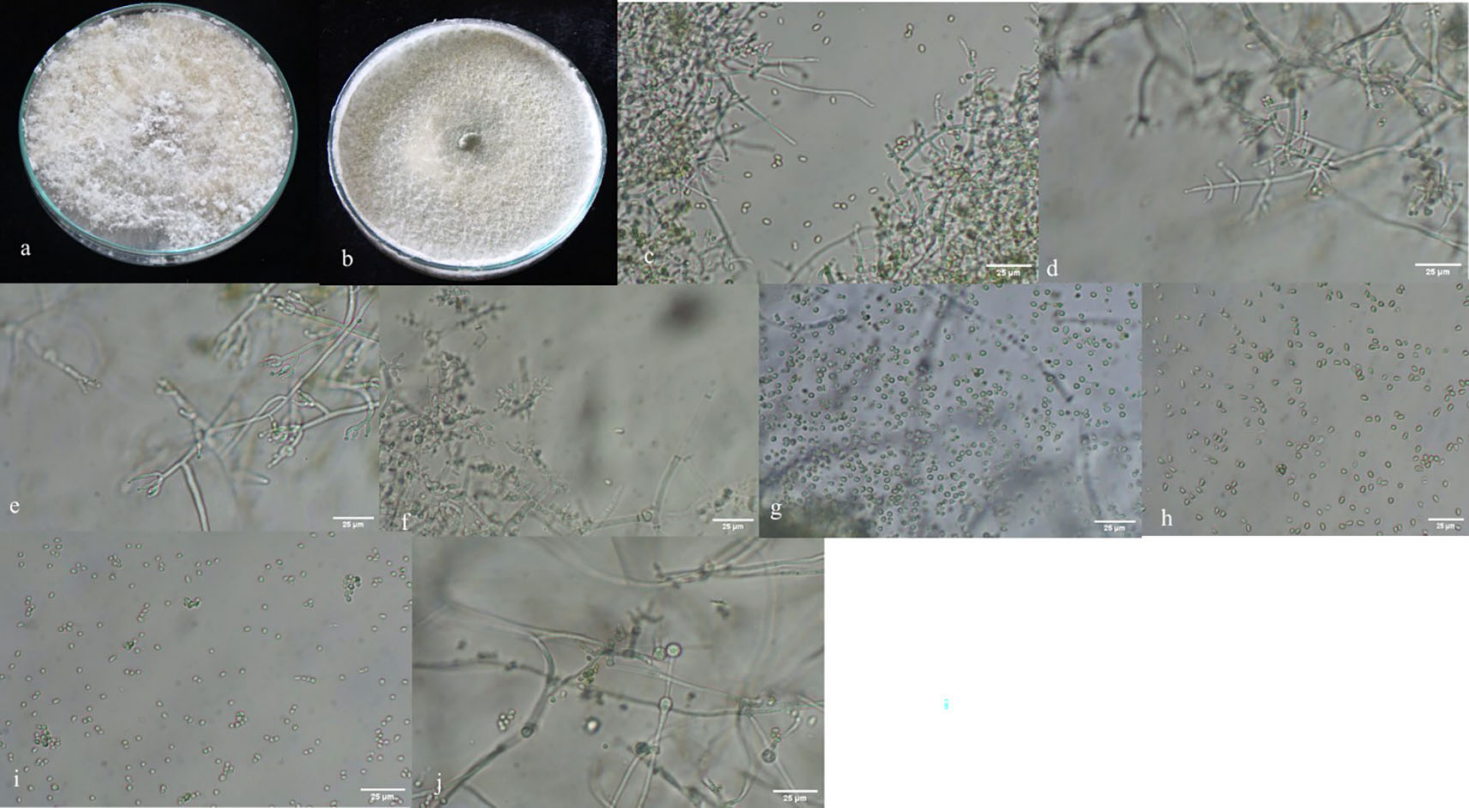
Morphological characteristics of *Trichoderma* isolates. a, b: with and without aerial mycelium. c–f: different types of phialides. g–i: different types of spores. j: presence of chlamydospores in *Trichoderma asperellum.*

A total of 167 *Trichoderma* isolates were isolated from soil samples collected from rhizospheric regions of various vegetable crops. Among this collection, 33 *Trichoderma* isolates were selected based on morphological and microscopic characteristics. The isolates were visually characterized based on phenotypic traits such as colony color (ranging from dark green to cottony whitish green, Plate 1), ^58^ growth pattern, presence or absence of aerial mycelium ( Figure 3a, b), as well as the shape and size of phialides ( Figure 3c–f) and conidia ( Figure 3g–i). Additionally, the presence ( Figure 3j) or absence of chlamydospores was observed microscopically ( Table 1). ^59^ Isolates from the groups T2, T3, T6, T7, T8, T9, T11, T14, T18, T19, T24 were morphologically and microscopically identified as belonging to the *Harzianum* clade. Meanwhile, isolates from groups T1, T4, T10, T12, T13, T15, T16, T21, T22, T23, T25 were identified as members of the *Viride* clade. Isolates from groups T5 and T17 were grouped within *Longibrachiatum* clade, corresponding to *T. longibrachiatum* species, while isolates from T20 group were classified within *Brevicompactum* clade, associated with *T. brevicompactum* species.

**Table 1.** Morphological characteristics of *Trichoderma* isolates isolated from various ecological zones of Nepal.

Name of *Trichoderma* isolates		Colony			Phialides			Conidia				Species	Molecular clade
		Form and color	Front color	Reverse color	Aerial mycelium	Conidiation	Size (μm)	Shape	Spore width (μm) ^x^	Coconut odor	Chlamydospore		
** T1**	** ToT8**	Green color concentric rings	Green	Banana yellow	Present	Whorl	5.8-9.5	Round	1.9-3.3	Present	Absent	*T. asperellum*	*Viride*
**T2**	**ST49**	green in middle and wooly white periphery	Green	Banana yellow	Absent	Whorl	5-8.5	Round	1.6-2.2	absent	Absent	*T. lentiforme*	*Harzianum*
**T3**	**BaT5**	Green and white ring	Fern green	Banana yellow	Absent	Whorl	5.1-6.6	Oblong	1.2-2.6	Absent	Absent	*T. afroharzianum*	*Harzianum*
**T4**	**ToT7**	Creamy wooly green colony	green	Cream	Absent	Whorl	5.0-7.0	Round	1.4-2.2	Present	Absent	*T. asperelloides*	*Viride*
**T5**	**PT11**	Green and white colony	green	White	Present	Whorl	4.9-7.4	Oblong	2-3.3/1.2-1.9	present	Present	*T. longibrachiatum*	*Longibrachiatum*
**T6**	**DT7**	Granular granny smith apple	Granny smith apple	cream	Absent	Whorl	6.1-8	Round	1.4-2.9	Absent	Present	*T. harzianum*	*Harzianum*
**T7**	**NaT2, MT5**	Concentric green ring colony	Green	Sunshine	Absent	Whorl	5-6.4	Round	1.9-2.6	Absent	Present	*T. afroharzianum, T. azevedoi*	*Harzianum*
**T8**	**MT8**	Concentric ring	Sea green	Straw	Absent	Whorl	4.9-7.4	Round	1.2-3.08	absent	Not clear	*T. inhamatum*	*Harzianum*
**T9**	**PanT1**	Sea green concentric ring	Sea green	Cream	Absent	Whorl	05-Sep	Oblong	2.5-4.1/0.9-1.9	Absent	Rarely Present	*T. camerunense*	*Harzianum*
**T10**	**ST43**	Fern green concentric ring	Fern green	Canary yellow	Present	Whorl	5-8.1	Oval	2.2-3.2/1.2-2.9	Present	Present	*T. asperellum*	*Viride*
**T11**	**ST10, SolT21, MT4, MT30, MT11, BaT9**	Fern green concentric ring	Fern green	Dijon pigment	Absent	Whorl	4.9-9.5	Round	0.9-1.9	Present	Rarely Present	*T. afroharzianum*	*Harzianum*
**T12**	**DT22**	White center and fern green	Fern green	Cream	Absent	Whorl	5.5-7.6	Round	1.02-2.17	Absent	Present	*T. koningii*	*Viride*
**T13**	**SaT4**	Granular fern green ring	Fern green	Banana yellow	Absent	Whorl	6.6-11.7	Round	1.2-2.9	Absent	Absen	*T. atroviride*	*Viride*
**T14**	**SolT24b**	Concentric granny smith apple colony	Granny smith apple green	Sand	Absent	Whorl	4.3-7.9	Round	.6-1.7	Absent	Present	*T. afroharzianum*	*Harzianum*
**T15**	**PanT2**	Granular green and white colony	Fern green	Tangerine	Absent	Whorl	5.8-10.4	Oblong	1.2-3.8/1.2-1.9	absent	Present	*T. asperelloides*	*Viride*
**T16**	**MT9**	Wooly white colony	Fern green	Pumpkin orange	Present	Whorl aggregate	6.3-8.8	Round	1.02-2.9	Absent	Rarely present	*T. asperellum*	*Viride*
**T17**	**ToT11**	Granular fern green colony	Fern green	Mustard yellow	Absent	Solitary	5.2-9.9	Oblong	2.3-3.6/0.9-1.9	Absent	Absent	*T. longibrachiatum*	*Longibrachiatum*
**T18**	**PT13**	Wooly white colony	White	Sunshine	Absent	Whorl	4.8-6.7	Oblong	2.5-3.6/.9-1.9	Present	Present	*T. afroharzianum*	*Harzianum*
**T19**	**ST6**	Green concentric ring	green	sunshine	Absent	Whorl	5.5-7.9	Round	.9-1.9	Absent	Present	*T. afroharzianum*	*Harzianum*
**T20**	**MT15**	Sea green ring colony	Sea green	straw	Absent	Whorl	7.7-10.6	Round	1.2-2.2	Absent	Present	*T. brevicompactum*	*Brevicompactum*
**T21**	**KT11, ST20**	Fern green colony	Fern green	Banana yellow	Absent	Whorl	7-9.6	Round	1.9-4	Absent	Present	*T.asperellum*	*Viride*
**T22**	**NaT7**	Fern green and white granular colony	Fern green	Canary yellow	Absent	2-3in Whorl	6.2-8.8	Round	1.9-3.3	Absent	Rarely Present	*T.asperellum*	*Viride*
**T23**	**KT6, SolT6b**	Granular green and white colony	Fern green	Cream base	Absent	2-3 in Whorl	6.8-8.7	Round	1.6-3.3	Present	Present	*T. asperellum*	*Viride*
**T24**	**ST3**	Wooly green colony	Green	Canary yellow	Absent	Solitary	6.2-7.7	Round	1.3-2	Absent	Present	*T. afroharzianum*	*Harzianum*
**T25**	**KT13**	Granular green and white colony	Fern green	white	Absent	Solitary/2-3whorl	5.5-8.4	Round	1.2-2.9	Absent	Present	*T. asperellum*	*Viride*

Morphological variations were observed among different groups of *Trichoderma* isolates ( Table 1). ^59^ Illustrations of colonies grown in PDA Petri plates are shown in Plate 1. ^58^ Various diffusible pigments were observed, displaying different colors, which were compared to the Audrey & Bear Color Chart ( https://audreyandbear.com/products/audrey-bear-color-chart). In the *Harzianum* clade, which includes species such as *T.* *afroharzianum*, *T. lentiforme*, *T. camerunense, T. inhamatum* and *T. azevedoi* colonies exhibited 1-2 concentric rings with the production of green-colored conidia *.* No diffusible pigments were detected in PDA medium, and a coconut odor was also not noticeable. The conidia were oval to oblong and ranged from 0.6-4.1μm. Within the *Viride clade*, comprising species like *T. asperellum, T. asperelloides, T. koningii* and *T. atroviride* colonies on PDA displayed a somewhat granular appearance with green and white regions distributed throughout the plate. No diffusible pigments were detected in the PDA medium, but coconut odor was found in some groups and absent in others ( Table 1). ^59^ The conidia were generally round and ranged from 1-4 μm. The T5 and T17 groups of isolates displayed white mycelium with green pustules and produced yellow diffusible pigments, classifying them within the *Longibrachiatum* clade. Conidia in this group were oblong and ranged from 2-3.9 μm. Isolates from T20 group were challenging to differentiate and were grouped together, with conidia measuring 1.2-2.2μm. Phialides were flask-shaped in both the *Harzianum* and *Viride* clades and were present in whorls in almost all species, except in ST3 ( *T. afroharzianum*) and ToT11( *T. longibrachiatum*), where they were solitary.

The *Harzianum* clade was found within an altitudinal range of 80-1800 masl, spanning from lowland plains to hilly regions, and was common in both tropical and temperate regions ( Table 2). ^59^ Similarly, the *Viride* clade were also identified in both tropical and temperate regions, with altitudes ranging from between 90-1475 masl, which was generally lower than that of the *Harzianum* clade. Isolates of the T20 group ( *T. brevicompactum*) were primarily located in lowland plains (at 80 masl), rather than in higher mountain region, whereas *T. longibrachiatum* was primarily found in mid-hill regions, with altitudes ranging from 1300-1500 masl ( Table 2). ^59^ However, morphological characteristics alone proved insufficient for distinguishing between the various *Trichoderma* isolates. Therefore, molecular identification was required to differentiate among complex and overlapping *Trichoderma* isolates.

**Table 2.** Details of selected *Trichoderma* isolates from different altitudinal regions with their corresponding geographic locations, isolate names and GenBank accession numbers.

Species	Isolate	GPS location	Altitude (masl)	District	Gen bank Accession No.	
					ITS	tef-1α
*T. afroharzianum*	ST3	26.54N 87.12E	80	Sunsari	OR140790	OR567133
*T. afroharzianum*	ST6	26.7N 87.28E	95	Sunsari	OR140791	OR567134
*T. afroharzianum*	ST10	26.58N 87.18E	113	Sunsari	OR140792	OR567135
*T. afroharzianum*	MT4	26.6N 87.32E	82	Morang	OR140793	OR567136
*T. afroharzianum*	MT11	26.46N 87.34E	100	Morang	OR140794	OR567137
*T. afroharzianum*	MT30	26.48N 87.27E	80	Morang	OR140795	OR567138
*T. afroharzianum*	BaT5	27.69N 85.25E	1360	Kathmandu	OR140796	OR567139
*T. afroharzianum*	BaT9	27.69N85.25E	1312	Kathmandu	*-*	OR567140
*T. afroharzianum*	NaT2	27.65N 85.51E	1605	Kavrepalanchok	OR140797	OR567141
*T. afroharzianum*	PT13	27.59N 85.51E	1510	Kavrepalanchok	*-*	OR567142
*T. afroharzianum*	SolT24b	27.44N 86.59E	1800	Solukhumbu	OR140798	OR567143
*T. lentiforme*	ST49	26.69N 87.09E	80	Sunsari	OR140799	OR567144
*T. inhamatum*	MT8	26.56N 87.49E	127	Morang	OR140800	OR567145
*T. camerunense*	PanT1	27.63N 85.55E	975	Kavrepalanchok	OR1407801	OR567146
*T. asperellum*	ST20	26.65N 87.28E	113	Sunsari	OR1407802	OR567147
*T. asperellum*	ST43	26.55N 87.12E	92	Sunsari	OR1407803	OR567148
*T. asperellum*	MT9	26.66N 87.6E	100	Morang	OR1407804	OR567149
*T. asperellum*	KT13	27.66N 85.29E	1413	Kathmandu	OR1407805	OR567150
*T. asperellum*	ToT7	27.75N 85.34E	1339	Kathmandu	OR1407806	OR567151
*T. asperellum*	ToT8	27.77N 85.33E	1339	Kathmandu	OR1407807	OR567152
*T. asperelloides*	PanT2	27.63N 85.61E	925	Kavrepalanchok	OR1407808	OR567153
*T. asperelloides*	KT11	27.68N 85.27E	1370	Kathmandu	*-*	OR567154
*T. atroviride*	SaT4	27.64N 85.48E	1475	Kavrepalanchok	*-*	OR567155
*T. brevicompactum*	MT15	26.49N 87.29E	80	Morang	OR140809	OR567156
*T. azevedoi*	MT5	26.49N 87.29E	100	Morang	OR140810	OR567157
*T. harzianum*	DT7	27.68N 85.93E	2500	Sindhupalchok	OR140811	*-*
*T. afroharzianum*	SolT21	27.54N 86.57E	2300	Solukhumbu	OR140812	*-*
*T. asperellum*	KT6	27.68N 85.27E	1335	Kathmandu	OR140813	*-*
*T. asperellum*	NaT7	27.65N85.51E	1624	Kavrepalanchok	OR140814	*-*
*T. asperellum*	SolT6b	27.52N 86.58E	2365	Solukhumbu	OR140815	*-*
*T. koningii*	DT22	27.68N 85.93E	2500	Sindhupalchok	OR140816	*-*
*T. longibrachiatum*	ToT11	27.75N 85.33E	1349	Kathmandu	OR140817	*-*
*T. longibrachiatum*	PT11	27.59N 85.51E	1520	Kavrepalanchok	OR140818	OR567158

### Molecular characterization and phylogenetic studies of *Trichoderma* isolates

*Trichoderma* isolates were further identified using molecular sequencing data of ITS rRNA and tef-1α genes to support our morphological data. Out of 33 strains, PCR and bidirectional sequencing were successfully completed for 29 presumptive isolates by ITS oligonucleotide barcode identification (OR140790-OR140818) and for only of 26 isolates using tef-1α (OR567133-OR567158). The average consensus sequence length for tef-1α was 663 bp, while for the ITS rRNA, it was 646 bp. Nucleotide BLAST analysis and pairwise alignments of the ITS rRNA and tef-1α sequences identified the *Trichoderma* isolates as belonging to *T. harzianum, T. afroharzianum, T. lentiforme, T. inhamatum, T. camerunense, T. azevedoi*, *T. atroviride, T. asperellum, T. asperelloides, T. brevicompactum, T. koningii and T. longibrachiatum* which shared 97-100% identity match with published sequences in the NCBI database.

**Figure 4.**
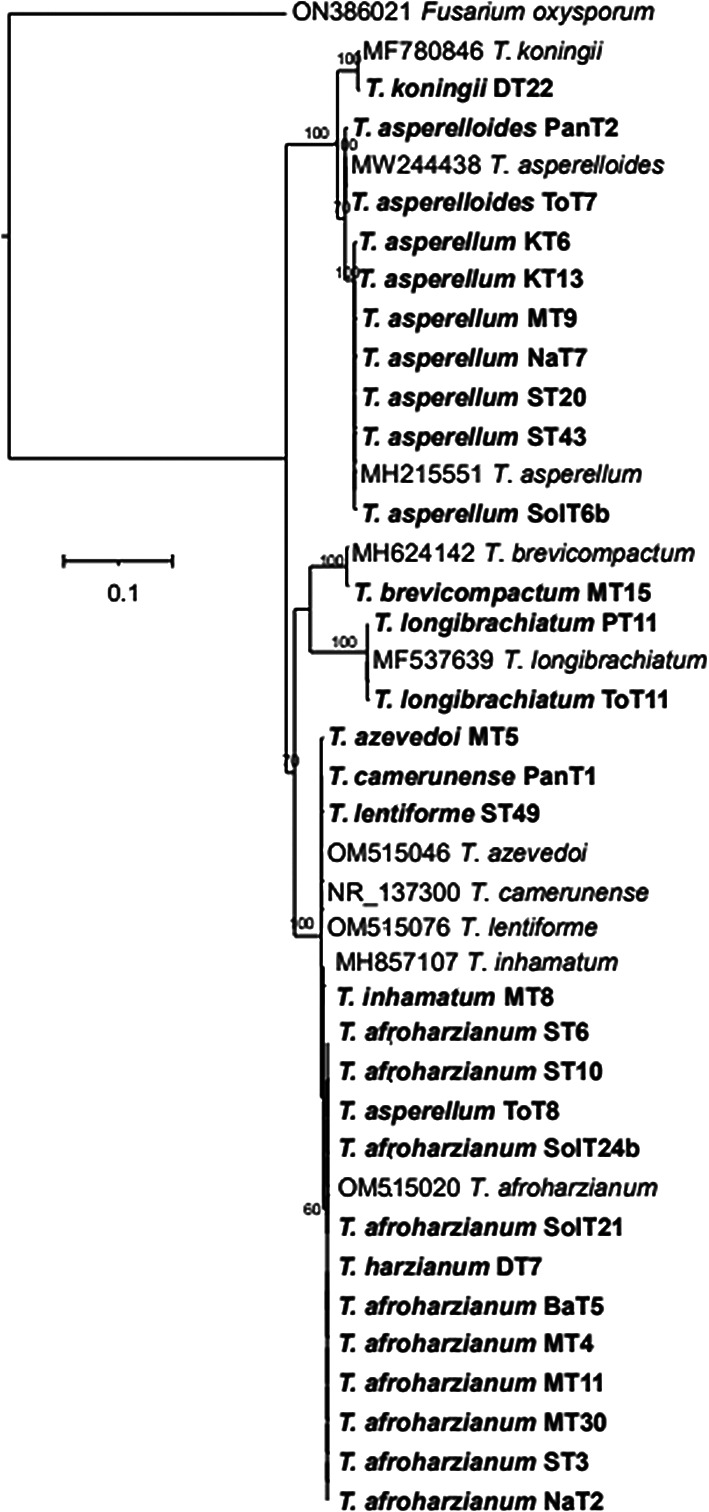
Phylogenetic tree of 29 *Trichoderma* isolates based on ITS rRNA sequences.

**Figure 5.**
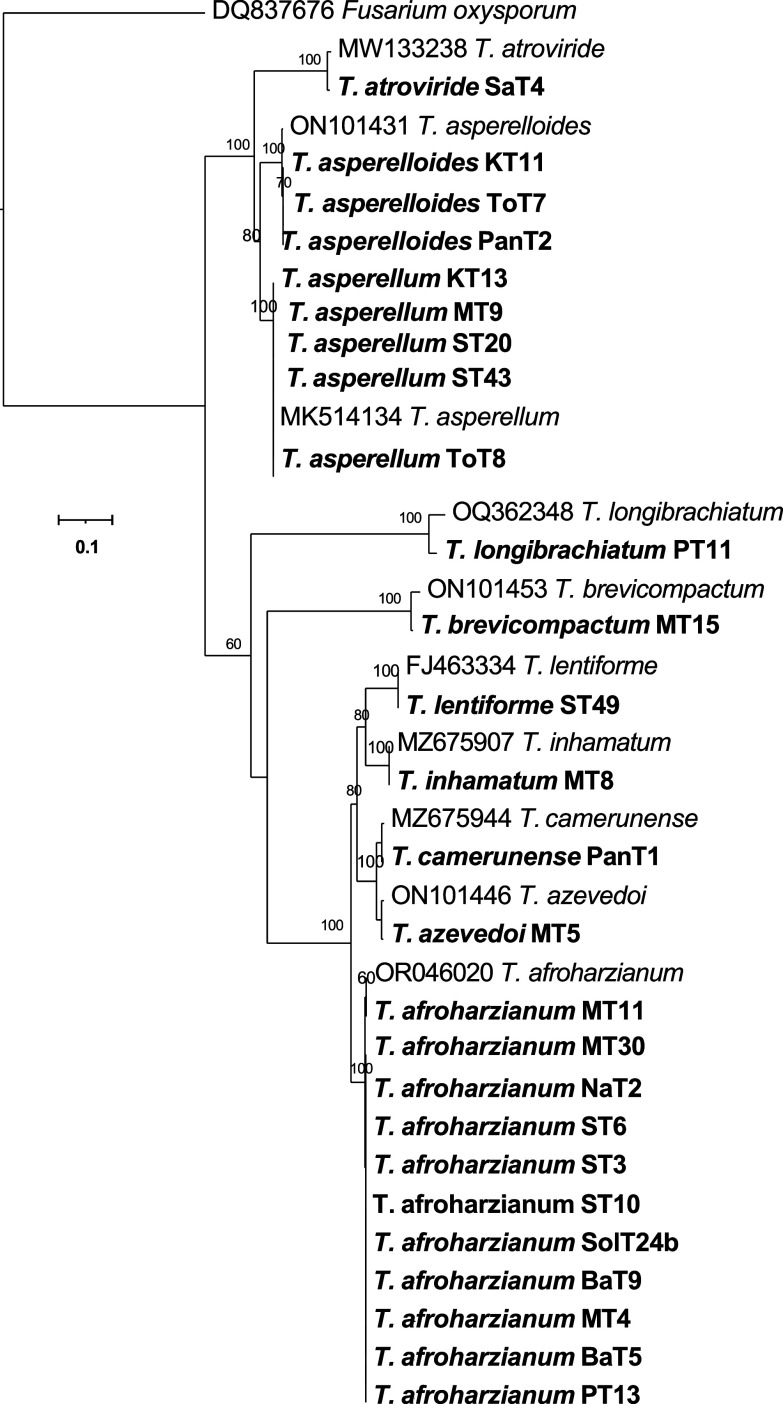
Phylogenetic tree of 26 *Trichoderma* isolates based on tef-1α gene sequences.

**Figure 6.**
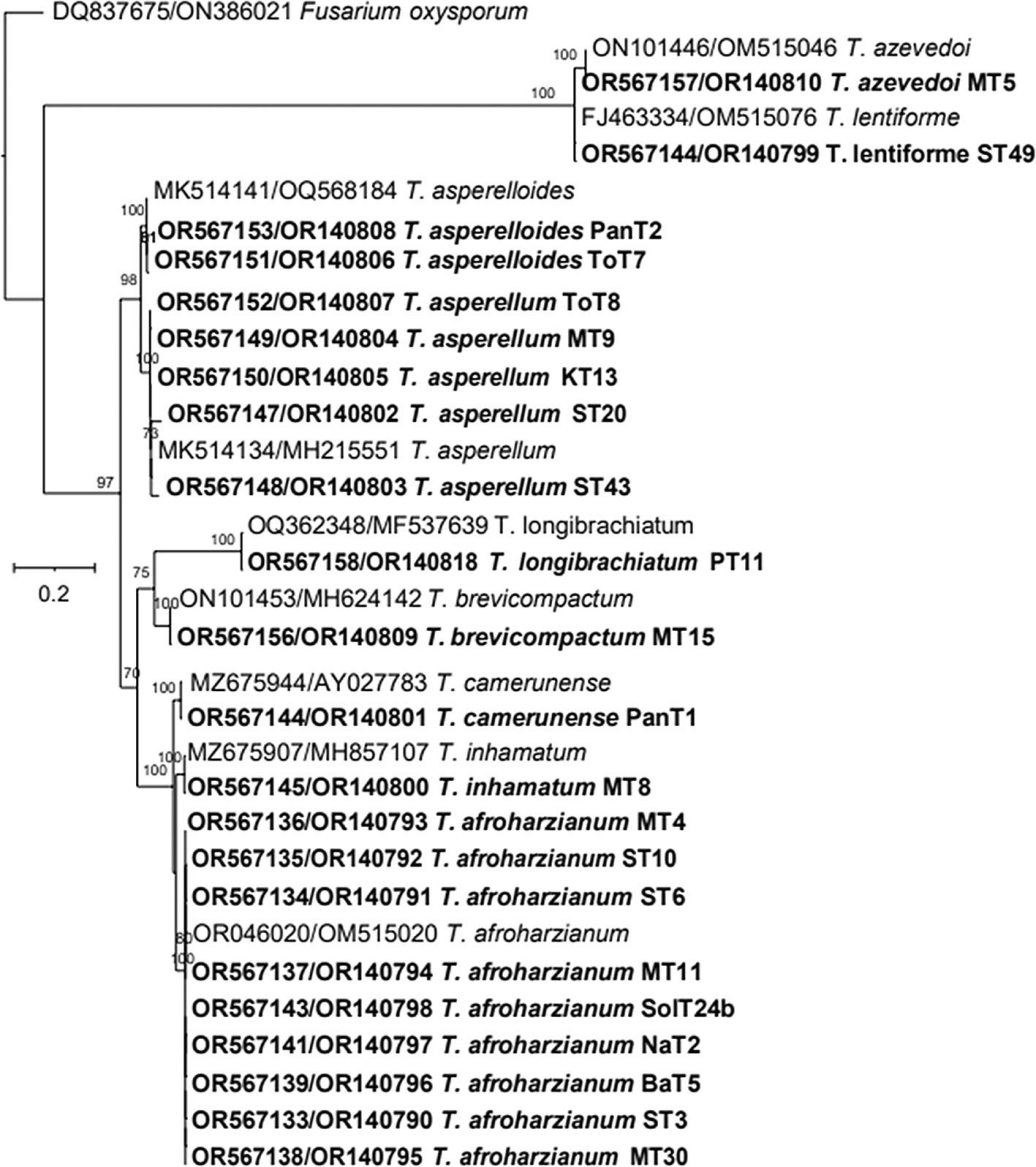
Phylogenetic tree of 22 *Trichoderma* isolates based on tef-1α -ITS gene sequences.

The phylogenetic tree ( Figure 4) based on ITS rRNA unambiguously identified 11 species among the 29 isolates, based on their clustering with reference taxa. The isolates identified in the analysis included *T. harzianum, T. afroharzianum, T. lentiforme, T. inhamatum, T. camerunense, T. azevedoi, T. asperellum, T. asperelloides, T. brevicompactum, T. koningii and T. longibrachiatum.* Although the ITS rRNA tree could not clearly delimit the species resolution of the isolates MT8, ST49, MT5, and PanT1, however there were clear and distinct resolution of other isolates ( Figure 4). The phylogenetic tree ( Figure 5) obtained from the tef-1α region identified 10 species among the 26 isolates, which included *T. afroharzianum, T. lentiforme, T. inhamatum, T. camerunense, T. azevedoi*, *T. atroviride, T. asperellum, T. asperelloides, T. longibrachiatum* and *T. brevicompactum.* The maximum likelihood phylogenetic tree, based on the concatenated dataset ITS and tef-1α (1352 bp), produced four distinct, well supported clades with bootstrap values higher than 70%: *Harzianum*, *Viride*, *Longibrachiatum* and *Brevicompactum* clades ( Figure 6).

Thus, based on above three phylogenetic trees, four clades were identified: *Harzianum*, *Viride*, *Brevicompactum* and *Longibrachiatum* clades. Eighty-five isolates belonged to the *Harzianum* clade, consisting of *T.* *afroharzianum* (64 isolates), *T. harzianum* (3 isolates), *T. lentiforme* (1 isolate), *T. camerunense* (11 isolates *T. inhamatum* (5 isolates), and *T. azevedoi* (1 isolate). The *Viride* clade included 67 isolates, comprising species such as *T.* *asperellum* (57 isolates), *T.* *asperelloides* (6 isolates), *T. atroviride* (1 isolate), and *T.* *koningii* (3 isolates). The *Longibrachiatum* clade comprised 11 isolates, all belonging to the *T. longibrachiatum.* The *Brevicompactum* clade consisted of 4 isolates, all of which were identified as *T. brevicompactum* ( Table 3). ^59^ Among these, *T. afroharzianum* was the most abundant species in this study, followed by *T. asperellum.*

**Table 3.** List of total number of *Trichoderma* isolates used for morphological characterization.

S. No.	Code group	No. of isolates present in code group	Name of all isolates in code group	Number of representative isolates taken	Isolates name taken for molecular characterization	Species name
1	T1	3	ToT8, NaT6, ST18	1	ToT8	*T. asperellum*
2	T2	1	ST49	1	ST49	*T. lentiforme*
3	T3	3	ST7, BaT5, MT39	1	BaT5	*T. afroharzianum*
4	T4	5	SaT7, ToT7, SolT3, SolT25, ST39a	1	ToT7	*T. asperelloides*
5	T5	6	ST38, PanT4, PT11, ToT9, PT14, ST8a	1	PT11	*T. longibrachiatum*
6	T6	3	LaT9, DT7, SolT10	1	DT7	*T. harzianum*
7	T7	14	NaT2, MT31, SaT6, NaT1, NaT5, ST2 NaT3, MT33, ST49a, SolT12a, ST9, SaT2, MT18, MT5	2	NaT2, MT5	*T. afroharzianum, T. azevedoi*
8	T8	5	ToT6, SolT26a, ST11, MT8, ToT4	2	MT8	*T. inhamatum*
9	T9	11	ST40, MT28, MT29, DT19, SolT6a, ST13, PanT1, DolT14, ToT1, ST27, KT2	1	PanT1	*T. camerunense*
10	T10	7	KT1, ST5, ST46, KT7, ST8b, ST39b, ST43	1	ST43	*T. asperellum*
11	T11	31	MT2, MT3, BKT4, MT46, BktT8, MT13, SaT1, ToT5, ST24, ST21, BaT9, SaT9, ST25, SolT14a, ST28, MT1, MT4, MT6, PT4, ST22, MT11, MT49, MT30, MT12, ST10, ST23, MT23, SolT21, MT37, MT19, MT48	6	SolT21, ST10, MT30, MT4, BaT9, MT11	*T. afroharzianum*
12	T12	3	ST1, PT6, DT22	1	DT22	*T. koningii*
13	T13	1	SaT4	1	SaT4	*T. atroviride*
14	T14	1	SolT24b	1	SolT24b	*T. afroharzianum*
15	T15	1	PanT2	1	PanT2	*T. asperelloides*
16	T16	1	MT9	1	MT9	*T. asperellum*
17	T17	5	PanT6, ST48, ST38, ST19, ToT11	1	ToT11	*T. longibrachiatum*
18	T18	7	PT13, DT24, DT31, DT26, SolT22, ST50, ST15	1	PT13	*T. afroharzianum*
19	T19	6	LaT3, ST12, KT12, ST6, ST4, ST23	1	ST6	*T. afroharzianum*
20	T20	4	MT10, MT14, MT17, MT15	1	MT15	*T. brevicompactum*
21	T21	27	BaT6, BaT12, ST26, LaT11, DT10, BaT3, MT44, PanT5, DT20, DT11, LaT4, PT5, NaT9, DT39, SolT7, MT24, SolT26b, DolT3, SaT3, ST20, SolT15, DolT23, BktT1, KT11, PanT9, SolT12b, DolT2	3	ST20, KT11	*T. asperellum*
22	T22	6	ST47, KT4, NaT7, SolT18, ST35, PanT8	1	NaT7	*T. asperellum*
23	T23	12	MT21, KT6, SolT6b, BktT5, BktT11, ST34, BktT3, DolT18, SolT13, SolT12c, BaT11, LaT12	2	KT6, SolT6b	*T. asperellum*
24	T24	3	BaT1, ST3, MT41	1	ST3	*T. afroharzianum*
25	T25	1	KT13	1	KT13	*T. asperellum*
Total	N = 25	N = 167		N = 33		

**Analysis of the diversity among** ***Trichoderma*** **species**

Table 4 shows Simpson’s biodiversity index (D), Shannon’s biodiversity index (H), evenness (J), and the abundance index (E) for various agricultural fields in different ecological zones. The highest species diversity and evennesswere recorded in the high mountain region, with Shannon’s index (H) of 1.724, evenness (J) of 0.84 and Simpson’s index (D) of 0.28). Following this the hilly region which exhibited the Shannon’s index of 1.563, eveness of 0.7 and Simpson’s index of 0.28. In contrast, the plain region had the lowest species diversity with Shannon’s and Simpson’s diversity indices and evenness estimated to be H = 1.515, J=0.66, D = 0.33. The species abundance values were E = 2.11 for the plain, E = 1.95 for mid-hill and E = 1.99 for the high mountain region. The dominance values (Y) of *T. afroharzianum T. asperellum*, *T. camerunense* and *T. longibrachiatum* were 0.04, 0.03, 0.01, and 0.01 respectively and they were classified as the principal species. On average, the diversity values for *Trichoderma* species were H = 1.61, D = 0.29, J = 0.74, and E= 2.02 ( Table 4). Simpson’s index (1-D) and the evenness index were close to 1 in all regions except for the plain region, indicating a very high diversity of *Trichoderma* species in the major agricultural areas of Nepal. The number of species and isolates, as well as the dominant species of *Trichoderma*, varied geographically ( Table 5) revealing that the mid hill and high mountain regions have a high diversity of *Trichoderma* species.

**Table 4.** Univariate diversity indices of *Trichoderma* isolates from different altitudinal region of Nepal.

Ecological indices	Ecological regions			
	Plain	Mid-hill	High mountain	Average
Simpson’s index (D)	0.33 (1-D = 0.67)	0.28 (1-D = 0.72)	0.28 (1-D = 0.72)	0.29
Shannon’s index (H)	1.515	1.563	1.724	1.61
Pielou's evenness (J)	0.66	0.72	0.84	0.74
Abundance index (E)	2.11	1.95	1.99	2.02

**Table 5.** Details of *Trichoderma* isolates from different altitudinal regions.

*Trichoderma* Species	Number of *Trichoderma* isolates			
	Plain	Mid-hill	High altitude	Total
*Trichoderma arfoharzianum*	38	18	8	64
*Trichoderma asperellum*	15	26	16	57
*Trichoderma camerunense*	5	3	3	11
*Trichoderma longibrachiatum*	4	6	1	11
*Trichoderma asperelloides*	1	3	2	6
*Trichoderma inhamatum*	2	2	1	5
*Trichoderma brevicompactum*	4	0	0	4
*Trichoderma harzianum*	0	1	2	3
*Trichoderma koningii*	1	1	1	3
*Trichoderma lentiforme*	1	0	0	1
*Trichoderma azevedoi*	1	0	0	1
*Trichoderma atroviride*	0	1	0	1
Total	72	61	34	167

## Discussion

The remarkable biodiversity found in Nepal can be attributed directly to its unique geographical position, which spans a broad spectrum of altitudinal variances and a wide array of climatic conditions. Consequently, Nepal holds a significant position as one of the world’s important biodiversity hotspots. The fungal genus *Trichoderma* has been extensively studied for its diverse metabolites and their applications in agriculture, industry and health. ^6^ Its ability to establish beneficial interactions with plants and produce bioactive compounds makes it a valuable resource for sustainable and eco-friendly solutions in multiple sectors. ^28^ However, there have been very few studies on the diversity of *Trichoderma* species in Nepal compared to other parts of the world. ^10^^,^^14^^,^^39^

In the present study, the isolates of *Trichoderma* (n = 167) isolated from soils of different ecological zones were categorized into three clades based on their morphological characterization: *Harzianum*, *Viride* and *Longibrachiatum.* However, some isolates were challenging to differentiate and were not assigned to any specific clade (T20). Our observations in terms of morphological characterization align with the previous studies on these species. ^42^^,^^60^ The use of a single method for identifying *Trichoderma* species can present challenges and potential issues. To address this concern, Bissett proposed integration of both morphological and molecular studies. ^61^ This combined approach allows for a more comprehensive and accurate species-level classification within *Trichoderma* spp. In our study, species were identified following previous studies that utilized ITS and tef-1α sequencing. ^62^ Using molecular approaches, we were able to clearly identify some cryptic species, and we also distinguished isolates that could not be identified solely based on morphological features.

*Trichoderma* species are typically identified through gene sequence analysis, with the generally accepted practice being the use of two gene sequences. However, recent studies suggest that the identification of new *Trichoderma* species may require the analysis of at least three DNA gene sequences. ^63^^,^^64^ In this particular study, 33 *Trichoderma* isolates were selected from a pool of 167 isolates isolated from soil samples that were collected from various regions of Nepal. To identify these isolates, we analyzed two DNA gene sequences: the ITS rRNA and the tef-1α genes. This analysis involved comparing the obtained sequences with those available in the NCBI database using BLASTn analysis. It has been observed that relying solely on individual gene sequences, such as ITS rRNA or tef-1α , is insufficient for accurately distinguishing all *Trichoderma* species.

The phylogenetic analysis using ITS rRNA and tef-1α regions revealed significant insights into the diversity and taxonomy of *Trichoderma* isolates. The ITS tree identified 11 species among 29 isolates, clustering most with reference taxa; however, it could not resolve species for four isolates (MT8, ST49, MT5, and PanT1), indicating its limitations for closely related species. In contrast, the tef-1α tree identified 10 species among 26 isolates, offering better resolution, particularly for closely related taxa such as *T. atroviride* and *T. asperelloides*. The maximum likelihood tree, based on a concatenated dataset (ITS + tef-1α), provided the most robust results, forming four well-supported clades: *Harzianum*, *Viride*, *Longibrachiatum*, and *Brevicompactum*. This approach demonstrated the enhanced resolution achieved by integrating multiple markers, confirming species boundaries and phylogenetic relationships. These findings emphasize the complementary roles of ITS and tef-1α in species identification and highlight the importance of combined datasets for accurate classification. Future work can expand on these results using whole-genome sequencing to further refine taxonomy and explore functional diversity.

As a result, we used a combination of ITS rRNA and tef-1α gene sequences, for a more comprehensive understanding of *Trichoderma* species distribution. In BLASTn searches, ITS sequences did not provide sufficient resolution of *Trichoderma* phylogeny for some species, such as the *T. harzianum* complex. However, the concatenated dataset (ITS-tef-1α) produced four well-supported clades. The resulting tree grouped together the same or closely related species into distinct clades. The comparison of test and conference taxa aligned with earlier reports. ^13^^,^^65^^,^^66^ The findings revealed similarities among *Trichoderma* species, with the out-group sequence of *Fusarium oxysporum* forming dissimilar clusters. Although tef-1α sequencing provided a clear picture of *Trichoderma* phylogeny, the predominant species complex ( *T. harzianum* complex) comprises multiple cryptic species. ^67^^,^^68^ The *T. harzianum* complex has undergone significant revision in recent times, resulting in the classification of *T. harzianum* into 14 distinct species. ^69^^,^^70^

In this research, we conducted a comprehensive examination of the diversity of indigenous *Trichoderma* species found in the rhizospheric soil across different districts of Nepal, taking into account variations in altitude. We assessed *Trichoderma* species diversity, which is defined as the product of the evenness and the number of species, using the Shannon biodiversity index -H. ^55^ To evaluate the dominance of individual species, we calculated Simpson’s diversity index, ^54^ which indicates the probability that two randomly selected species from a given ecosystem will belong to different categories. We also used Margalef’s abundance index to assess species richness and the Pielou index to determine the evenness of the *Trichoderma* population. The diversity and occurrence of *Trichoderma* species reported from different agroclimatic zones of Nepal, namely, the plain (80-141 masl), mid-hill (1284-1650 masl), and high mountain (1800-2500 masl) regions, clearly indicate that climatic topography and soil type are major factors influencing the distribution of *Trichoderma* species.

The present study aimed to assess the suitably of several widely used diversity indices for various types of statistical analyses. We conducted both simple and multifaceted statistical analyses to determine if certain indices were better suited for specific types of analyses. Notably, Simpson’s index(1-D) and the evenness index approached 1 in the mid-hill and high mountain regions, except in the plain region. This observation suggests a remarkably high diversity of *Trichoderma* species in the major agricultural areas of Nepal, particularly in the mid-hill and high mountain regions. This is obvious that mountains and hills have high climatic diversity due to latitudinal variation compared to plain and harbor higher diversity of *Trichoderma* species compared to plain.

These findings underscore the substantial diversity of *Trichoderma* species in these specific regions. Interestingly, Shannon’s index yielded quite similar values (~1.5) in the plain and mid-hill regions, which could be attributed to the extensive disturbance caused by human activities in both regions. The results of this study indicate that *Trichoderma* spp. diversity and habitat preference can serve as natural indicators of rhizosphere soil health. Moreover, our findings align with the research conducted by Ma ^53^ in Chinese grasslands of China and Mulatu ^71^ in Ethiopian coffee ecosystems. This diversity analysis of *Trichoderma* strains will facilitate the improved identification of *Trichoderma* species with biocontrol mechanisms, which can, in turn, contribute to the development of suitable bioformulations in sustainable agriculture.

The number of *Trichoderma* species and isolates, as well as the dominant species, varied significantly across different geographical regions. The plain region (80-141 masl) encompasses two districts, Sunsari and Morang. In this region, we identified a total of six different species of *Trichoderma*: *T. afroharzianum, T. lentiforme, T. inhamatum, T. asperellum, T. brevicompactum* and *T. azevedoi.* We found a total of 72 isolates in the plain region, with *T. afroharzianum* (38 isolates) being the dominant species. The mid-hill region (1284-1650 masl) includes four main districts: Kathmandu, Lalitpur, Bhaktapur, and Kavrepalnchok. Here we identified six different types of *Trichoderma* species: *T. afroharzianum, T. camerunense*, *T* *. longibrachiatum, T. asperellum, T. asperelloides* and *T. atroviride* with total of 61 isolates. In the mid-hill region, *T. asperellum* (26 isolates) was the dominant species. The high mountain region (1800-2500 masl) comprises two districts, Solukhumbu and Sindhupalchok. In this region, we found only four *Trichoderma* species: *T. harzianum, T. afroharzianum, T. asperellum* and *T. koningii*, with a total of 34 isolates. In this region, *T. asperellum* (16 isolates) was the dominant species. The presence of these *Trichoderma* species in the rhizospheric soils of vegetables in Nepal can be attributed to the diverse ecological substrates and climate conditions in the country’s vegetable crop growing areas. Our results suggest that these areas provide an optimal environment for the survival and colonization of a diverse group of *Trichoderma* species. *Trichoderma afroharzianum* (39.0%) was found to be the most widely distributed and abundant species in our study. The occurrence of *Trichoderma* species is influenced by various factors, including metabolic diversity, reproductive capabilities, substrate availability and the competitive abilities of *Trichoderma* isolates in natural ecosystems.

In the present, we identified nine new country record of *Trichoderma* species: *T. afroharzianum T. harzianum, T. lentiforme, T. inhamatum, T. camerunense, T. atroviride, T. brevicompactum, T. longibrachiatum* and *T. azevedoi.* However, these species have previously been described in regions across the world, including America, ^68^^,^^72^^,^^73^Asia, ^10^^,^^16^^,^^17^Africa ^9^^,^^35^^,^^71^and European Mediterranean countries. ^25^^,^^46^ During this study, we reisolated some species, including *T. asperellum*, *T. asperelloides* and *T. koningii*, along with nine additional species. However, two previously reported species, *T. rugulosum* and *T. lixii* were not found in this study. ^10^^,^^39^ The *Harzianum* clade contained economically important species such as *T. harzianum, T. afroharzianum, T. camerenunse, T. lentiforme* and *T. inhamatum*, which are used in agriculture as biological control agents. The *Viride* clade included *T. asperellum, T. asperelloides*, *T. koningii* and *T. atroviride.* The *Longibrachiatum* clade consisted of *T. longibrachiatum*, while the *Brevicompactum* clade with *T. brevicompactum.* *T. longibrachiatum* has high optimal and maximum growth temperatures and exhibits yellow reverse pigmentation due to the production of secondary metabolites such as pyrone. *T. brevicompactum* is utilized in the production of various antimicrobial substances, offering significant agricultural, health, and environmental benefits.

In this study, we obtained isolates of *T. harzianum*, *T. afroharzianum*, *T. inhamatum*, *T. lentiforme*, *T. azevedoi*, *T. camerunense, T. asperellum*, *T.* *asperelloides*, *T. atroviride*, *T. koningii*, *T. longibrachiatum*, and *T. brevicompactum* from various crop ecosystems. Notably, *T. afroharzianum* and *T. asperellum* were most widely distributed species in our study. However, previous studies in crop ecosystems in Nepal have reported *T. asperellum* and *T. asperelloides* as the most widely distributed species of this genus. ^39^ It is worth noting that among these reported species, only *T.* *asperellum*, *T. asperelloides* and *T. koningii* have been previously documented in Nepal. ^10^^,^^39^

## Conclusion

This study presents novel findings regarding the presence of *Trichoderma* species in the Himalayan foothills of Nepal at different altitudes in previously unexplored geographic regions. However, it is important to note that our observations provide only a limited glimpse into the overall diversity of *Trichoderma* in Nepal. Despite being recognized as a biodiversity hotspot, fungal diversity in this region remains poorly understood. To gain a more comprehensive understanding, further investigations involving the isolation of *Trichoderma* from various substrates like forest areas and high-altitude areas in Nepal are highly recommended. Such endeavors are likely to unveil a multitude of additional species, some of which may be previously unknown. These new discovered taxa hold significant potential for various biotechnological applications and can contribute to further advancements in this field.

## Author information

**Authors and Affiliations**

**Puja Jaiswal**

Central Department of Zoology, Tribhuvan University, Kirtipur, Nepal

**Ram B. Khadka and Suraj Baidya**

National Plant Pathology Research Center, Nepal Agricultural Research Council, Khumaltar, Nepal

**Aashaq Hussain Bhat**

Department of Bioscience, University Centre for Research and Development, Chandigarh University, Punjab, India

**Arvind Kumar Keshari**

Department of Zoology, Patan Multiple Campus, Tribhuvan University, Lalitpur, Nepal

## Author’s contributions

**Puja Jaiswal** conceptualized, did data curation, formal analysis, investigation, methodology, validation and writing original draft. **Ram Bahadur Khadka** worked on conceptualization, formal analysis, investigation, methodology, project administration, resources, supervision, and validation of the study. **Suraj Baidya** helped with writing, review and editing the manuscript. **Aashaq Hussain Bhat** did formal analysis, validation and writing, review and editing the manuscript. **Arvind Kumar Keshari** conceptualization, project administration, resources, supervision, validation, writing, review and editing the manuscript. All authors read and agree to the final draft of the paper.

## Ethics and consent

The present study didn’t involve the use of human and animals so ethical approval and consent were not required.

## Data Availability

The sequencing data are available on the NCBI Genbank webpage **Morphological and Molecular Characterization of Native *Trichoderma* isolates from Nepal** (OR140790- OR140818; OR567133-OR567158) NCBI: *Trichoderma* Internal Transcribed Spacer RNA (ITS): Accession number OR140790; https://www.ncbi.nlm.nih.gov/nuccore/OR140790 ^59^ NCBI: *Trichoderma* Internal Transcribed Spacer RNA (ITS): Accession number OR140791; https://www.ncbi.nlm.nih.gov/nuccore/OR140791 ^59^ NCBI: *Trichoderma* Internal Transcribed Spacer RNA (ITS): Accession number OR140792; https://www.ncbi.nlm.nih.gov/nuccore/OR140792 ^59^ NCBI: *Trichoderma* Internal Transcribed Spacer RNA (ITS): Accession number OR140793; https://www.ncbi.nlm.nih.gov/nuccore/OR140793 ^59^ NCBI: *Trichoderma* Internal Transcribed Spacer RNA (ITS): Accession number OR140794; https://www.ncbi.nlm.nih.gov/nuccore/OR140794 ^59^ NCBI: *Trichoderma* Internal Transcribed Spacer RNA (ITS): Accession number OR140795; https://www.ncbi.nlm.nih.gov/nuccore/OR140795 ^59^ NCBI: *Trichoderma* Internal Transcribed Spacer RNA (ITS): Accession number OR140796; https://www.ncbi.nlm.nih.gov/nuccore/OR140796 ^59^ NCBI: *Trichoderma* Internal Transcribed Spacer RNA (ITS): Accession number OR140797; https://www.ncbi.nlm.nih.gov/nuccore/OR140797 ^59^ NCBI: *Trichoderma* Internal Transcribed Spacer RNA (ITS): Accession number OR140798; https://www.ncbi.nlm.nih.gov/nuccore/OR140798 ^59^ NCBI: *Trichoderma* Internal Transcribed Spacer RNA (ITS): Accession number OR140799; https://www.ncbi.nlm.nih.gov/nuccore/OR140799 ^59^ NCBI: *Trichoderma* Internal Transcribed Spacer RNA (ITS): Accession number OR140800; https://www.ncbi.nlm.nih.gov/nuccore/OR140800 ^59^ NCBI: *Trichoderma* Internal Transcribed Spacer RNA (ITS): Accession number OR1407801; https://www.ncbi.nlm.nih.gov/nuccore/OR1407801 ^59^ NCBI: *Trichoderma* Internal Transcribed Spacer RNA (ITS): Accession number OR1407802; https://www.ncbi.nlm.nih.gov/nuccore/OR1407802 ^59^ NCBI: *Trichoderma* Internal Transcribed Spacer RNA (ITS): Accession number OR1407803; https://www.ncbi.nlm.nih.gov/nuccore/OR1407803 ^59^ NCBI: *Trichoderma* Internal Transcribed Spacer RNA (ITS): Accession number OR1407804; https://www.ncbi.nlm.nih.gov/nuccore/OR1407804 ^59^ NCBI: *Trichoderma* Internal Transcribed Spacer RNA (ITS): Accession number OR1407805; https://www.ncbi.nlm.nih.gov/nuccore/OR1407805 ^59^ NCBI: *Trichoderma* Internal Transcribed Spacer RNA (ITS): Accession number OR1407806; https://www.ncbi.nlm.nih.gov/nuccore/OR1407806 ^59^ NCBI: *Trichoderma* Internal Transcribed Spacer RNA (ITS): Accession number OR1407807; https://www.ncbi.nlm.nih.gov/nuccore/OR1407807 ^59^ NCBI: *Trichoderma* Internal Transcribed Spacer RNA (ITS): Accession number OR1407808; https://www.ncbi.nlm.nih.gov/nuccore/OR1407808 ^59^ NCBI: *Trichoderma* Internal Transcribed Spacer RNA (ITS): Accession number OR140809; https://www.ncbi.nlm.nih.gov/nuccore/OR140809 ^59^ NCBI: *Trichoderma* Internal Transcribed Spacer RNA (ITS): Accession number OR140810; https://www.ncbi.nlm.nih.gov/nuccore/OR140810 ^59^ NCBI: *Trichoderma* Internal Transcribed Spacer RNA (ITS): Accession number OR140811; https://www.ncbi.nlm.nih.gov/nuccore/OR140811 ^59^ NCBI: *Trichoderma* Internal Transcribed Spacer RNA (ITS): Accession number OR140812; https://www.ncbi.nlm.nih.gov/nuccore/OR140812 ^59^ NCBI: *Trichoderma* Internal Transcribed Spacer RNA (ITS): Accession number OR140813; https://www.ncbi.nlm.nih.gov/nuccore/OR140813 ^59^ NCBI: *Trichoderma* Internal Transcribed Spacer RNA (ITS): Accession number OR140814; https://www.ncbi.nlm.nih.gov/nuccore/OR140814 ^59^ NCBI: *Trichoderma* Internal Transcribed Spacer RNA (ITS): Accession number OR140815; https://www.ncbi.nlm.nih.gov/nuccore/OR140815 ^59^ NCBI: *Trichoderma* Internal Transcribed Spacer RNA (ITS): Accession number OR140816; https://www.ncbi.nlm.nih.gov/nuccore/OR140816 ^59^ NCBI: *Trichoderma* Internal Transcribed Spacer RNA (ITS): Accession number OR140817; https://www.ncbi.nlm.nih.gov/nuccore/OR140817 ^59^ NCBI: *Trichoderma* Internal Transcribed Spacer RNA (ITS): Accession number OR140818; https://www.ncbi.nlm.nih.gov/nuccore/OR140818 ^59^ NCBI: *Trichoderma* DNA sequence of translation elongation factor 1α (tef-1): Accession number OR567133; https://www.ncbi.nlm.nih.gov/nuccore/OR567133 ^59^ NCBI: *Trichoderma* DNA sequence of translation elongation factor 1α (tef-1α): Accession number OR567134; https://www.ncbi.nlm.nih.gov/nuccore/OR567134 ^59^ NCBI: *Trichoderma* DNA sequence of translation elongation factor 1α (tef-1α): Accession number OR567135; https://www.ncbi.nlm.nih.gov/nuccore/OR567135 ^59^ NCBI: *Trichoderma* DNA sequence of translation elongation factor 1α (tef-1α): Accession number OR567136; https://www.ncbi.nlm.nih.gov/nuccore/OR567136 ^59^ NCBI: *Trichoderma* DNA sequence of translation elongation factor 1α (tef-1α): Accession number OR567137; https://www.ncbi.nlm.nih.gov/nuccore/OR567137 ^59^ NCBI: *Trichoderma* DNA sequence of translation elongation factor 1α (tef-1α): Accession number OR567138; https://www.ncbi.nlm.nih.gov/nuccore/OR567138 ^59^ NCBI: *Trichoderma* DNA sequence of translation elongation factor 1α (tef-1α): Accession number OR567139; https://www.ncbi.nlm.nih.gov/nuccore/OR567139 ^59^ NCBI: *Trichoderma* DNA sequence of translation elongation factor 1α (tef-1α): Accession number OR567140; https://www.ncbi.nlm.nih.gov/nuccore/OR567140 ^59^ NCBI: *Trichoderma* DNA sequence of translation elongation factor 1α (tef-1α): Accession number OR567141; https://www.ncbi.nlm.nih.gov/nuccore/OR567141 ^59^ NCBI: *Trichoderma* DNA sequence of translation elongation factor 1α (tef-1α): Accession number OR567142; https://www.ncbi.nlm.nih.gov/nuccore/OR567142 ^59^ NCBI: *Trichoderma* DNA sequence of translation elongation factor 1α (tef-1α): Accession number OR567143; https://www.ncbi.nlm.nih.gov/nuccore/OR567143 ^59^ NCBI: *Trichoderma* DNA sequence of translation elongation factor 1α (tef-1α): Accession number OR567144; https://www.ncbi.nlm.nih.gov/nuccore/OR567144 ^59^ NCBI: *Trichoderma *DNA sequence of translation elongation factor 1α (tef-1α): Accession number OR567145; https://www.ncbi.nlm.nih.gov/nuccore/OR567145 ^59^ NCBI: *Trichoderma *DNA sequence of translation elongation factor 1α (tef-1α): Accession number OR567146; https://www.ncbi.nlm.nih.gov/nuccore/OR567146 ^59^ NCBI: *Trichoderma* DNA sequence of translation elongation factor 1α (tef-1α): Accession number OR567147; https://www.ncbi.nlm.nih.gov/nuccore/OR567147 ^59^ NCBI: *Trichoderma* DNA sequence of translation elongation factor 1α (tef-1α): Accession number OR567148; https://www.ncbi.nlm.nih.gov/nuccore/OR567148 ^59^ NCBI: *Trichoderma* DNA sequence of translation elongation factor 1α (tef-1α): Accession number OR567149; https://www.ncbi.nlm.nih.gov/nuccore/OR567149 ^59^ NCBI: *Trichoderma* DNA sequence of translation elongation factor 1α (tef-1α): Accession number OR567150; https://www.ncbi.nlm.nih.gov/nuccore/OR567150 ^59^ NCBI: *Trichoderma* DNA sequence of translation elongation factor 1α (tef-1α): Accession number OR567151; https://www.ncbi.nlm.nih.gov/nuccore/OR567151 ^59^ NCBI: *Trichoderma* DNA sequence of translation elongation factor 1α (tef-1α): Accession number OR567152; https://www.ncbi.nlm.nih.gov/nuccore/OR567152 ^59^ NCBI: *Trichoderma* DNA sequence of translation elongation factor 1α (tef-1α): Accession number OR567153; https://www.ncbi.nlm.nih.gov/nuccore/OR567153 ^59^ NCBI: *Trichoderma* DNA sequence of translation elongation factor 1α (tef-1α): Accession number OR567154; https://www.ncbi.nlm.nih.gov/nuccore/OR567154 ^59^ NCBI: *Trichoderma* DNA sequence of translation elongation factor 1α (tef-1α): Accession number OR567155; https://www.ncbi.nlm.nih.gov/nuccore/OR567155 ^59^ NCBI: *Trichoderma* DNA sequence of translation elongation factor 1α (tef-1α): Accession number OR567156; https://www.ncbi.nlm.nih.gov/nuccore/OR567156 ^59^ NCBI: *Trichoderma* DNA sequence of translation elongation factor 1α (tef-1v): Accession number OR567157; https://www.ncbi.nlm.nih.gov/nuccore/OR567157 ^59^ NCBI: *Trichoderma* DNA sequence of translation elongation factor 1α (tef-1α): Accession number OR567158; https://www.ncbi.nlm.nih.gov/nuccore/OR567158 ^59^ Figshare: Morphological and molecular characterization of *Trichoderma* isolates from vegetable crop rhizospheres in Nepal; Plate 1. Culture of *Trichoderma* isolates grown in 28±2°c in potato dextrose agar medium; https://doi.org/10.6084/m9.figshare.26405059.v1 ^58^ Data are available under the terms of the Creative Commons Attribution 4.0 International license (CC-BY 4.0) Figshare: Morphological and molecular characterization of *Trichoderma* isolates from vegetable crop rhizospheres in Nepal; https://doi.org/10.6084/m9.figshare.26380360.v1 ^59^ Data are avalilable under the terms of the Creative Commons Attribution 4.0 Internasional license (CC-BY 4.0)
