# Supplementary material for: Morphological and Molecular Characterization of Trichoderma Isolates from Vegetable Crop Rhizospheres in Nepal
Source: F1000Res. 2025 Mar 5;13:1088. Originally published 2024 Sep 24. [Version 3] doi: 10.12688/f1000research.153701.3 (PMC11773134; doi:10.12688/f1000research.153701.3)
Supplement: Peer review history

# Reviewer report: Reviewer response for version 2

**DOI:** 10.5256/f1000research.176431.r357675

**Published:** 2025-01-27

**Authors:** Yunus Korkom

**Affiliations:** 1 Aydın Adnan Menderes University, Aydin, Turkey

I have no new comments in this article.

Is the work clearly and accurately presented and does it cite the current literature?

Yes

If applicable, is the statistical analysis and its interpretation appropriate?

Not applicable

Are all the source data underlying the results available to ensure full reproducibility?

Yes

Is the study design appropriate and is the work technically sound?

Yes

Are the conclusions drawn adequately supported by the results?

Yes

Are sufficient details of methods and analysis provided to allow replication by others?

Yes

Reviewer Expertise:

Trichoderma, plant pathology, biological control, soil-borne diseases, fruit diseases, endophytic fungi, fungal pathogens,

I confirm that I have read this submission and believe that I have an appropriate level of expertise to confirm that it is of an acceptable scientific standard.

# Reviewer report: Reviewer response for version 2

**DOI:** 10.5256/f1000research.176431.r359924

**Published:** 2025-01-24

**Authors:** Eder Marques

**Affiliations:** 1 Federal University of Goiás, Campus Samambaia, Goiânia, Brazil

The work expands the knowledge about the diversity of *Trichoderma *in Nepal, however some suggestions were made to improve the study.

**Abstract **– diversify repeated keywords

**Introduction**

 Some references do not relate to the idea for which they were cited. It would be interesting to replace them (check observations in the text)

 Describe a little more the genus Trichoderma and its importance for the world and Nepal. Are there data on the quantity of commercial products and their use, in form, for example, in the country?

**Methodology**

 Clarify the criteria used for selecting representative isolates from the initial pool of 167 isolates. This will improve reproducibility.

 I was unable to find information regarding the plants from which the rhizospheric soil samples were collected.

 Morphological and cultural characteristics are not usable characteristics in the identification of Trichoderma, perhaps try to concatenate and discuss with molecular phylogeny.

 Baysian inference should be done, in addition to ML.

 Which equipment is used at that temperature (-40ºC)?

 Provide more detail about the statistical analysis software and versions used for diversity index calculations.

**Results**

 Concatenation of morphological and molecular information.

 Figure 3. Does it mention different types of conidia, what about them? Shape, size? Describe!

 Plate 1: Unclear photos, fungus does not appear to have sporulated (no color).

 Figure 4: Expand the tree, it is not possible to visually differentiate the branches/clades.

 Figure 6. Describe a little better in the caption. Concatenated phylogeny?

 Correct italics in inappropriate places.

 The work expands the knowledge about the diversity of Trichoderma in Nepal, however some suggestions were made to improve the study.

Is the work clearly and accurately presented and does it cite the current literature?

No

If applicable, is the statistical analysis and its interpretation appropriate?

I cannot comment. A qualified statistician is required.

Are all the source data underlying the results available to ensure full reproducibility?

Yes

Is the study design appropriate and is the work technically sound?

Partly

Are the conclusions drawn adequately supported by the results?

Yes

Are sufficient details of methods and analysis provided to allow replication by others?

Partly

Reviewer Expertise:

Prospecting for Trichoderma for biological control.

I confirm that I have read this submission and believe that I have an appropriate level of expertise to confirm that it is of an acceptable scientific standard, however I have significant reservations, as outlined above.

## References

[1] : Population density of Trichoderma fungi in natural environments and agrosystems of a Cerrado area. *Biota Neotropica* .2020;20(4) : 10.1590/1676-0611-bn-2020-1048 10.1590/1676-0611-bn-2020-1048

[2] : New isolates of Trichoderma antagonistic to Sclerotinia sclerotiorum. *Biota Neotropica* .2016;16(3) : 10.1590/1676-0611-BN-2016-0218 10.1590/1676-0611-BN-2016-0218

# Response

**Published:** 2025-02-18

**Authors:** Ram Khadka

**Affiliations:** Plant Pathology, Nepal Agricultural Research Council, Kathmandu, Bagmati, Nepal

**Reviewer 3**

 The work expands the knowledge about the diversity of *Trichoderma *in Nepal, however some suggestions were made to improve the study.

 Response: We sincerely appreciate your time and effort in reviewing our manuscript and for providing insightful suggestions to improve its quality.

**Abstract **– diversify repeated keywords

 Response: Thank you for your suggestion. However, "diversity indices" is a technical term essential to maintaining the accuracy and context of our study. Replacing it with alternative terms might alter the intended meaning, so we have retained it.

**Introduction**

 Some references do not relate to the idea for which they were cited. It would be interesting to replace them (check observations in the text)

 Describe a little more the genus Trichoderma and its importance for the world and Nepal. Are there data on the quantity of commercial products and their use, in form, for example, in the country?

 Response: We appreciate this valuable feedback. We have carefully reviewed and updated the references to ensure their relevance. Additionally, we have expanded the introduction on the importance of *Trichoderma*, in Nepal, including available data on its commercial applications in the country.

**Methodology**

 Clarify the criteria used for selecting representative isolates from the initial pool of 167 isolates. This will improve reproducibility.

 Response: Thank you for your suggestion. We have now included a clear explanation in the first paragraph of the "Molecular Characterization of *Trichoderma* Isolates" subsection, detailing the criteria used for selecting 33 isolates for molecular characterization.

 I was unable to find information regarding the plants from which the rhizospheric soil samples were collected.

 Response: The soil samples were collected from the rhizosphere of ten different crop species. This information has been clearly stated in the Methodology section.

 Morphological and cultural characteristics are not usable characteristics in the identification of Trichoderma, perhaps try to concatenate and discuss with molecular phylogeny.

 Response: We acknowledge the reviewer's concern. While molecular techniques provide a more precise identification, morphological characteristics, despite their limitations, remain valuable for selecting superior-performing isolates. We have revised the discussion to better integrate morphological data with molecular phylogeny.

 Baysian inference should be done, in addition to ML.

**Response: **We appreciate the suggestion regarding Bayesian inference. Upon analysis, we observed that both the Maximum Likelihood (ML) and Bayesian Inference (BI) trees exhibit highly similar topologies, with no significant differences in clade support or branching patterns. However, if the reviewer suggests additional statistical comparisons or specific analyses, we would be happy to incorporate them.

 Which equipment is used at that temperature (-40ºC)?

 Response: The isolates were stored in a deep freezer at -40°C. This has now been clarified in the text.

 Provide more detail about the statistical analysis software and versions used for diversity index calculations.

 Response: The biological diversity indices were calculated numerically using the provided equations in Microsoft Excel (Excel 2010, v14.0). This information has been explicitly stated in the manuscript.

**Results**

 Concatenation of morphological and molecular information.

 Response: We appreciate this suggestion and have improved the integration of morphological and molecular data to enhance clarity and consistency in our findings.

 Figure 3. Does it mention different types of conidia, what about them? Shape, size? Describe!

 Response: Yes, Figure 3 depicts different types of conidia. Their shape and size are detailed in Table 1.

 Plate 1: Unclear photos, fungus does not appear to have sporulated (no color).

 Response: We put both upside and inverted plates together to show the variations in conidial color, metabolite production and presence or absence of aerial mycelia.

 Figure 4: Expand the tree, it is not possible to visually differentiate the branches/clades.

 Response: We appreciate the suggestion and have revised Figures 4 and 5 to enhance clarity and improve branch differentiation.

 Figure 6. Describe a little better in the caption. Concatenated phylogeny?

 Response: We have refined the caption while adhering to the journal’s guideline of a 15-word limit.

 Correct italics in inappropriate places.

 Response: We have thoroughly reviewed the manuscript and corrected all instances of incorrect italicization.

 The work expands the knowledge about the diversity of Trichoderma in Nepal, however, some suggestions were made to improve the study.

 Response: We sincerely appreciate the reviewer’s thoughtful comments, which have helped us refine and improve the manuscript.

- **Is the work clearly and accurately presented and does it cite the current literature?**

No

- **Is the study design appropriate and is the work technically sound?**

Partly

- **Are sufficient details of methods and analysis provided to allow replication by others?**

Partly

- **If applicable, is the statistical analysis and its interpretation appropriate?**

I cannot comment. A qualified statistician is required.

- **Are all the source data underlying the results available to ensure full reproducibility?**

Yes

- **Are the conclusions drawn adequately supported by the results?**

Yes

**References**

 1. Silva J, Marques E, Menezes J, Silva J, et al.: Population density of Trichoderma fungi in natural environments and agrosystems of a Cerrado area. *Biota Neotropica*. 2020; **20** (4). Publisher Full Text

 2. Marques E, Martins I, Cunha M, Lima M, et al.: New isolates of Trichoderma antagonistic to Sclerotinia sclerotiorum. *Biota Neotropica*. 2016; **16** (3). Publisher Full Text

 Response: These above references are cited in the text as 12 and 31.

# Reviewer report: Reviewer response for version 1

**DOI:** 10.5256/f1000research.168625.r331288

**Published:** 2024-11-25

**Authors:** Raja Asad Ali Khan

**Affiliations:** 1 School of Breeding and Multiplication (Sanya Institute of Breeding and Multiplication), Hainan University, YaZhou, China

The manuscript provides insights into the diversity of Trichoderma species in agricultural zones across Nepal, integrating morphological and molecular approaches. The research is robust and presents significant findings with potential applications in sustainable agriculture. However, a few minor revisions are recommended to enhance clarity and overall quality.

 Expand briefly on the practical implications of Trichoderma spp. for small-scale farmers in Nepal, considering the local agricultural context.

 Clarify the criteria used for selecting representative isolates from the initial pool of 167 isolates. This will improve reproducibility.

 Provide more detail about the statistical analysis software and versions used for diversity index calculations.

 In Table 1, consider aligning the morphological features with molecular clades for easier interpretation.

 Expand the discussion on the discrepancies observed between ITS and tef-1α sequencing results, emphasizing their implications for species identification.

Is the work clearly and accurately presented and does it cite the current literature?

Yes

If applicable, is the statistical analysis and its interpretation appropriate?

Yes

Are all the source data underlying the results available to ensure full reproducibility?

Yes

Is the study design appropriate and is the work technically sound?

Yes

Are the conclusions drawn adequately supported by the results?

Yes

Are sufficient details of methods and analysis provided to allow replication by others?

Yes

Reviewer Expertise:

Mycology, Biocontrol

I confirm that I have read this submission and believe that I have an appropriate level of expertise to confirm that it is of an acceptable scientific standard, however I have significant reservations, as outlined above.

# Response

**Published:** 2024-12-28

**Authors:** Ram Khadka

**Affiliations:** Plant Pathology, Nepal Agricultural Research Council, Kathmandu, Bagmati, Nepal

Expand briefly on the practical implications of *Trichoderma* spp. for small-scale farmers in Nepal, considering the local agricultural context.

**Response: **We really appreciate your valuable time to read and provide highly constructive feedback to improve the manuscript. As per the suggestions, the practical implication of *Trichoderma* in small-scale farmers’ perspectives in Nepal has been added. Please check the fourth paragraph in the introduction section.

 Clarify the criteria used for selecting representative isolates from the initial pool of 167 isolates. This will improve reproducibility.

**Response**: Thank you for the suggestion, the sentence has been added to the first paragraph of the “Method” section in the “Molecular characterization of *Trichoderma* isolates” subsection to clarify the selection criteria for 33 isolates for molecular characterization.

 Provide more detail about the statistical analysis software and versions used for diversity index calculations.

**Response: **The biological diversity indices were computed numerically using the given equations in Microsoft Excel (Excel 2010 (v14.0)). This has been explained.

 In Table 1, consider aligning the morphological features with molecular clades for easier interpretation.

**Response: **Thank you for the comments, the species indicated in Table 1 are identified through molecular characterization, and the molecular clade group has been added to the table. Please check Table 1.

 Expand the discussion on the discrepancies observed between ITS and tef-1α sequencing results, emphasizing their implications for species identification.

**Response:** Thanks for the suggestions, a paragraph has been added in discussion section. Please see fourth paragraph in discussion section.

# Reviewer report: Reviewer response for version 1

**DOI:** 10.5256/f1000research.168625.r327961

**Published:** 2024-10-04

**Authors:** Yunus Korkom

**Affiliations:** 1 Aydın Adnan Menderes University, Aydin, Turkey

Dear Author,

 The article is well written. But it would be even better with some suggestions below.

 Best regards,

**Comments**

**Introduction**

 “These surveys have been conducted in various locations, including Russia and the Siberian Himalayas, ^10^, ^11^ China, ^12^, ^13^ South-East Asia, ^14 ^India, ^15^ Egypt, ^16^ Iran, ^17 ^Philippines, ^18^ Europe, ^19^ Canary Islands, ^20^ Sardinia ^21^and South America. ^22”^

**The following studies conducted in Turkey can be added here.**

- Korkom, Y., & Yıldız, A. (2024). First report of *Trichoderma guizhouense* isolated from soil in Türkiye. *Journal of Plant Diseases and Protection*, *131*(2), 619-625. 

“Certain *Trichoderma* species have been successfully utilized as BCAs, showcasing their antagonistic mechanisms to mitigate damage caused by soil-borne pathogens, both in greenhouse and open field conditions. ^25–27^”

- Korkom, Y., & Yildiz, A. (2022). Evaluation of biocontrol potential of native *Trichoderma *isolates against charcoal rot of strawberry. *Journal of Plant Pathology*, *104*(2), 671-682.

**Methods**

**Isolation and culture of *Trichoderma* isolates**

- “After inoculation, *Trichoderma* colonies were sub cultured onto potato dextrose agar (PDA) plates and incubated at 28°C for seven days to allow for development and growth.” **should** **replaced with **“After inoculation, *Trichoderma* colonies were sub cultured onto potato dextrose agar  (PDA) plates and incubated at 28°C for seven days to allow for growth.”

 

- “Colonies have key characteristics that can be used to identify them as Trichoderma including growth pattern, growth speed, **odour** and colour.”

**The odour is not a characteristic feature used to distinguish *Trichoderma *species and should be removed.**

  

- “the Audrey & Bear Color Chart (https://www.audrey and bear.com)” **When the given link is opened in the browser, an irrelevant page opens.**

**Molecular characterization of *Trichoderma* isolates**

- “colonies cultured on potato dextrose agar (PDA) using sterilized” **should be replaced with **“colonies cultured on PDA using sterilized”

 

- “Promega Wizard genomic DNA extraction kit” **catalogue number** **should be added.**

 

- “the internal transcribed spacer” **should be replaced with **“the internal transcribed spacer (ITS)”

**Phylogenetic relationships**

  

- “in the NCBI” **should be replaced with **“in The National Center for Biotechnology Information (NCBI)”

**Results**

**Morphological characterization of single spores of *Trichoderma* isolates**

- “the Audrey & Bear Color Chart (https://www.audreyandbear.com)” **When the given link is opened in the browser, an irrelevant page opens.**
- “The maximum likelihood phylogenetic tree, based on the concatenated dataset ITS and tef-1α (1352 bp),” **Were the concatenated dataset of 26 isolates used here or 29 isolates? (Figure 6)**

Is the work clearly and accurately presented and does it cite the current literature?

Yes

If applicable, is the statistical analysis and its interpretation appropriate?

Not applicable

Are all the source data underlying the results available to ensure full reproducibility?

Yes

Is the study design appropriate and is the work technically sound?

Yes

Are the conclusions drawn adequately supported by the results?

Yes

Are sufficient details of methods and analysis provided to allow replication by others?

Yes

Reviewer Expertise:

Trichoderma, plant pathology, biological control, soil-borne diseases, fruit diseases, endophytic fungi, fungal pathogens,

I confirm that I have read this submission and believe that I have an appropriate level of expertise to confirm that it is of an acceptable scientific standard, however I have significant reservations, as outlined above.

## References

[1] : First report of Trichoderma guizhouense isolated from soil in Türkiye. *Journal of Plant Diseases and Protection* .2024;131(2) : 10.1007/s41348-023-00828-3 619-625 10.1007/s41348-023-00828-3

[2] : Evaluation of biocontrol potential of native Trichoderma isolates against charcoal rot of strawberry. *Journal of Plant Pathology* .2022;104(2) : 10.1007/s42161-022-01063-9 671-682 10.1007/s42161-022-01063-9

# Response

**Published:** 2024-12-27

**Authors:** Ram Khadka

**Affiliations:** Plant Pathology, Nepal Agricultural Research Council, Kathmandu, Bagmati, Nepal

**Comments**

**Introduction**

 “These surveys have been conducted in various locations, including Russia and the Siberian Himalayas, ^10^, ^11^ China, ^12^, ^13^ South-East Asia, ^14 ^India, ^15^ Egypt, ^16^ Iran, ^17 ^Philippines, ^18^ Europe, ^19^ Canary Islands, ^20^ Sardinia ^21^and South America. ^22”^

**The following studies conducted in Turkey can be added here.**

- Korkom, Y., & Yıldız, A. (2024). First report of *Trichoderma guizhouense* isolated from soil in Türkiye. *Journal of Plant Diseases and Protection*, *131*(2), 619-625. 

“Certain *Trichoderma* species have been successfully utilized as BCAs, showcasing their antagonistic mechanisms to mitigate damage caused by soil-borne pathogens, both in greenhouse and open field conditions. ^25–27^”

- Korkom, Y., & Yildiz, A. (2022). Evaluation of biocontrol potential of native *Trichoderma *isolates against charcoal rot of strawberry. *Journal of Plant Pathology*, *104*(2), 671-682.

**Response: As per the recommendation both the studies are cited as 23 ad 29 .**

**Methods**

**Isolation and culture of *Trichoderma* isolates**

- “After inoculation, *Trichoderma* colonies were sub cultured onto potato dextrose agar (PDA) plates and incubated at 28°C for seven days to allow for development and growth.” **should replaced with **“After inoculation, *Trichoderma* colonies were sub cultured onto potato dextrose agar (PDA) plates and incubated at 28°C for seven days to allow for growth.”

** Response: As per suggestion sentence is corrected.**

 “Colonies have key characteristics that can be used to identify them as Trichoderma including growth pattern, growth speed, **odour** and colour.”

**The odour is not a characteristic feature used to distinguish *Trichoderma *species and should be removed.**

**Response: We have removed odour as suggested.**

  

- “the Audrey & Bear Color Chart (https://www.audrey and bear.com)” **When the given link is opened in the browser, an irrelevant page opens.**

**Response:**  The link is corrected as https://audreyandbear.com/products/audrey-bear-color-chart

**Molecular characterization of *Trichoderma* isolates**

- “colonies cultured on potato dextrose agar (PDA) using sterilized” **should be replaced with **“colonies cultured on PDA using sterilized”

- “Promega Wizard genomic DNA extraction kit” **catalogue number** **should be added.**

 
**Response:**  We have added catalogue number as Catalogue No. A1120.

- “the internal transcribed spacer” **should be replaced with **“the internal transcribed spacer (ITS)”

**Phylogenetic relationships**

  

- “in the NCBI” **should be replaced with **“in The National Center for Biotechnology Information (NCBI)”

**Response: Both the above suggestions are incorporated.**

**Results**

**Morphological characterization of single spores of *Trichoderma* isolates**

- “the Audrey & Bear Color Chart (https://www.audreyandbear.com)” **When the given link is opened in the browser, an irrelevant page opens.**

**Response:** The link is corrected as https://audreyandbear.com/products/audrey-bear-color-chart

- “The maximum likelihood phylogenetic tree, based on the concatenated dataset ITS and tef-1α (1352 bp),” **Were the concatenated dataset of 26 isolates used here or 29 isolates? (Figure 6)**

**Response: **For the concatenation dataset of 22 isolates were used.
